# How to establish digital health ecosystems from the perspective of health service-organizations: A taxonomy developed based on expert interviews conducted as modified Delphi approach

**DOI:** 10.1177/20552076241271890

**Published:** 2024-08-08

**Authors:** Robin Huettemann, Benedict Sevov, Sven Meister, Leonard Fehring

**Affiliations:** 1 235785 Faculty of Health, School of Medicine, Witten/Herdecke University, Witten, Germany; 2 Healthcare Informatics, Faculty of Health, School of Medicine, Witten/Herdecke University, Witten, Germany; 3 Department Healthcare, 28476 Fraunhofer Institute for Software and Systems Engineering ISST, Dortmund, Germany; 4 Gastroenterology, HELIOS University Hospital Wuppertal, Witten/Herdecke University, Wuppertal, Germany

**Keywords:** Prevention, digital health, health communications, online, technology, connected care

## Abstract

**Objective:**

Digital health ecosystems may be the next revolution in improving citizens’ well-being, health delivery, data management, and health system processes, but solutions have not yet been broadly established. Reasons could be that health service-organizations have misaligned interests or lack capabilities. This study investigates reasons from a multi-health-service-organization perspective, differentiating between payers, insurers, healthcare providers, and innovators, detailing the expected value-adds, preferred participation roles, and required capabilities including a rating assessment.

**Methods:**

Findings are based on a taxonomy development methodology, which combines a literature review with semi-structured qualitative expert interviews, conducted using a modified Delphi approach. Interviews were thematically analysed.

**Results:**

In total, 21 experts across the four health service-organization groups were interviewed. The capability taxonomy includes a total of 16 capabilities, categorized in three themes: ‘Health market’, ‘organizational’, and ‘technology and informatic’. Providers expect a value-add from strengthening their health process economics through efficiency gains but reveal the largest capability gaps, especially in ‘interoperability’ and ‘platform’. Innovators’ ‘technology and informatic’ capabilities complement well with those of payers for the ‘health market’.

**Conclusions:**

We present a health service-organization-specific three-stage approach for establishing digital health ecosystems. Payers and insurers should address their ‘technology and informatic’ capability gaps, using technical enablers or forming new entities to reduce dependencies from legacy information technology systems. Innovators should clarify their monetization models and create positive awareness for their services, possibly entering the market directly. Providers must address interoperability issues and may require incentives to encourage their participation. Findings suggest governmental policymakers to prioritize three health policy initiatives.

## Introduction

Advances in internet use, informatics, and technologies, including electronic health (eHealth)^1^ and mobile health (mHealth),^2^ have transformed interactions in healthcare,^3,4^ and have been collectively summarized under the umbrella term digital health^5,6^ defined by the World Health Organization as ‘[…] development and use of digital technologies to improve health. Digital health expands the concept of eHealth to include digital consumers, with a wider range of smart-devices and connected equipment. It also encompasses other uses of digital technologies for health such as the Internet of things, artificial intelligence, big data and robotics’.^7^

Further advances around informatics and technologies, alongside the limited adaption, respectively adherence of citizens,^8^ have given rise to the concept of digital health ecosystems as the next revolution beyond digital health. They differentiate from often standalone and single-service focused digital health applications (apps) by integrating various digital and in-person health services, making them centrally accessible through a single web interface and/or mobile app for citizens.^9–13^ These integrated services can expand across the entire health journey, including prevention, diagnosis, treatment, and payment services.^14–17^ These services are typically provided by multiple health service-organizations, coordinated by one central orchestrator.^18–20^ All this eliminates the need for citizens to engage with multiple digital health apps to satisfy their health-related needs, thereby increasing convenience and potentially usage and adherence.^9,16,21^ Thus, digital health ecosystems are often referred to as the next revolution in improving the delivery of health, citizens’ well-being, data management, and health system processes.^12,22^ Based on these distinctions from digital health and derived through a combination of previously used descriptions,^9–13,20,23,24^ in this research study, digital health ecosystems are defined as follows:

Digital health ecosystems are citizen-facing online applications (apps) such as mobile apps and web interfaces, which integrate, complement, and facilitate access to various digital and in-person (professional-personal) health services along the health journey. Typically, these services are provided by one or multiple health service-organizations on the supply side (e.g., healthcare service-organizations’, public payers, private insurers). These health service-organizations share the common vision of improving citizens’ well-being while enabling the efficient delivery of health services and trusted interactions with citizens on the demand side. This is orchestrated by one health service-organization in the role of a leader, operating within the policies/regulations and data/technology infrastructure standards set by governmental policymakers.

From this definition, three involved stakeholder groups (levels) with distinct roles can be derived. Governmental policymakers’ (macro level) role is to establish health policies and regulatory boundaries, as well as standards that all stakeholders must adhere to. This exemplarily includes setting technology infrastructure standards, defining data structures, or granting market access approval. Health service-organizations (meso level) operate along the health journey to facilitate the supply of in-person and digital health-related services to citizens. This includes both public sponsored and for-profit organizations, which typically deliver health-related services through direct exchanges with citizens. Examples include healthcare providers (e.g. hospitals, local practitioners), public payers, and private insurers, among others. Additionally, health service-organizations can also play a less (non) citizen facing role by facilitating the delivery of health-related services. Examples of these include pharmaceuticals, hospital-information-system companies, among others. Citizens (micro level) play the role of demanders for health-related services throughout the health journey, and potentially act as social caregivers.^25^ Citizens as caregivers in a professional (clinical) context would classify under the meso level (health service-organizations, e.g. affiliated with a healthcare provider or care institution), and in a private context under the micro level (e.g. parents treating their children, or vice versa at home).

Each of the three stakeholder groups has different expectations regarding the advantages of digital health ecosystems. Citizens and health service-organizations may expect advantages from improved health data management, health system process efficiencies, and citizens’ well-being, including the promotion of prevention services, higher accuracies in diagnostics, and personalized treatments.^5,12,25–28^ Governmental policymakers may aim to address broader health system challenges, primarily the growing health supply gap and health system expenditures.^28–31^ This digitization shift toward digital health ecosystems may affect 90% of the health system, leading to profound cultural, social, and economic implications. This includes changes in the roles of stakeholders and requires new capabilities from them.^32^

To effectively implement supportive health policies/regulations and data/technology infrastructure standards, governmental policymakers must prioritize and sequence their efforts based on the perspectives of both health service-organizations and citizens, ensuring adaptability.^33,34^

Already prior to the COVID-19 pandemic, citizens downloaded 3.7 billion digital health apps annually, indicating a demand for digital health ecosystems.^35^ This demand has likely increased due to the COVID-19 pandemic,^9^ alongside a reduction in costs associated with enabling technologies, such as wearables and connecting devices.^36^ Exemplary, nowadays 40% of German citizens use one digital health or fitness app at least weekly.^37^ Yet, in most countries, this demand is mainly met by single-issue-focused digital health apps but has not been met with the supply of digital health ecosystems.^38^ This may diminish citizens’ perceived value and affect adherence, as they are required to engage with multiple non-integrated digital health apps while they increasingly seek to address and manage all their health needs centrally.^9,12,21,39^ This disparity between demand and supply points toward health service-organizations as the cause for the absence of broadly established digital health ecosystems.

In fact, while the perspective of citizens was the focus of previous research,^33^ the understanding of the health service-organizations’ perspectives remains limited for two reasons: first, most scientific data collections took place before the COVID-19 pandemic, neglecting recent changes. Second, their perspectives have not been systematically examined across distinct groups, ignoring their varying priorities and capabilities that potentially inhibit cooperation for ecosystem establishment.^33^

Therefore, the present study focuses on and differentiates between four distinct health market native and direct citizen facing health service-organization groups at the meso level to broaden the understanding of the health service-organizations’ perspectives: (1) public payers (payers), (2) private insurers (insurers), (3) healthcare providers (e.g. hospitals providing in-person, and potentially digital services) (providers),^33,40^ and (4) health innovators/start-ups (innovators).^41^ The focus on health market native health service-organizations ensures that findings are distinct to the characteristics of the health market. Innovators are in scope as they present a more technology and less health market legacy-driven perspective, while they offer a broad range of transformative digital health-related services, including, but not limited to, support for preventive lifestyles, self-treatment, data management, and appointments.^4,39^ They have primarily driven recent digital health innovations^4^ and account for the majority of available digital health apps.^39^ So-called ‘Big Tech’ or ‘Social Media’ companies (e.g. Facebook or Amazon) were not interviewed as a separate stakeholder group, although they begin to enter the health market.^42^ Yet, only Apple introduced ‘Apple Health’ as a meaningful offering. Previous research indicates a limited acceptance (beyond fitness tracking) by citizens for these companies. For example, citizens may be hesitant to use these companies’ apps to communicate with their practitioners,^43^ or to share their comprehensive health data records.^44^ Larger technology companies (e.g. hospital-information-system companies) are not investigated as a group, due to their limited consumer-facing role so far.

Considering the differences in health system structures across countries, previous research suggested country-specific approaches to establish digital health ecosystems.^9,25,45^ While recognizing this, still, this study aims at developing findings generalizable across countries worldwide. Therefore, the study sample focuses on Germany leveraging its dual health system, which integrates different health system structures used worldwide into a single health system, including public payers and private insurers as well as mandatory and supplementary insurances. Citizens can choose between public payers and private insurers for their mandatory health insurance, which reimburses for primary health market services, including diagnostics and treatments.^46–48^ While private health market economics are usually driven by higher prices but lower volume, the public health market operates vice versa, which creates an economic trade-off consideration for health service-organizations. For secondary health market services (e.g. preventive services), regulatory restrictions are lower, while insurance is supplementary but provided by private insurers only. In contrast to payers, insurers can tailor pricing and reimbursements to individual citizens based on their risk profiles.^49^ Hence, we are convinced that findings derived from a German study sample have relevance to other countries worldwide.

The objective of this study is to generate findings that support health service-organizations globally in the establishment of digital health ecosystems. Ultimately, these findings may enable governmental policymakers to prioritize their health policy/regulation and data/technology infrastructure standard setting. Previous research, albeit not focused on Germany, has indicated initial factors that may inhibit the establishment of digital health ecosystems by health service-organizations, including misaligned value-add expectations,^33^ a lack of clarity regarding roles,^50^ or deficiencies in capability readiness.^3,12,33,45^ Hence, the research question is three-fold:

What are the (1) expected value-adds, (2) preferred participation roles, and (3) required capabilities including potential capability gaps from the perspective of four distinct health service-organization groups, with regards to the establishment of digital health ecosystems?

## Methods

### Overview of three-step taxonomy development methodology

This study follows a three-step taxonomy development methodology, as outlined by Nickerson, Varshney, and Muntermann,^51^ to develop the final taxonomy, accompanied by a rating assessment. The three steps include an initial literature review to inform the following two steps of primary data collection through semi-structured qualitative expert interviews (referred to as interviews), using a modified Delphi approach.

Initially, a priori themes and codes for the initial taxonomy were derived based on a literature review.

Second, the first Delphi round of interviews facilitated the derivation of interim results, which included an iterative revision (detailing, renaming, or merging) of the initial taxonomy (‘conceptual-to-empirical’ (CtE)) and its supplementation (‘empirical-to-conceptual’ (EtC)),^51^ accompanied by an initial rating assessment.

Third, through additional interviews as part of the second Delphi round, the interim results were iteratively either confirmed, revised, or supplemented until the final taxonomy and rating assessment accurately reflected the interview data.^52^

Findings from the interview data were derived following a thematic analysis,^53^ satisfying the ‘subjective ending condition’, while the agreement definition of the Delphi approach fulfilled the ‘objective ending condition’ of the taxonomy development methodology.

The ‘Ethics Committee of the Witten/Herdecke University’ (No. S-265/2022) raised no objection regarding ethical concerns. Copyrights for figures, tools, the interview guide, and all other materials used were fully respected, and permissions were obtained for this research study if required.

### Literature review as secondary data source

**Figure 1.**
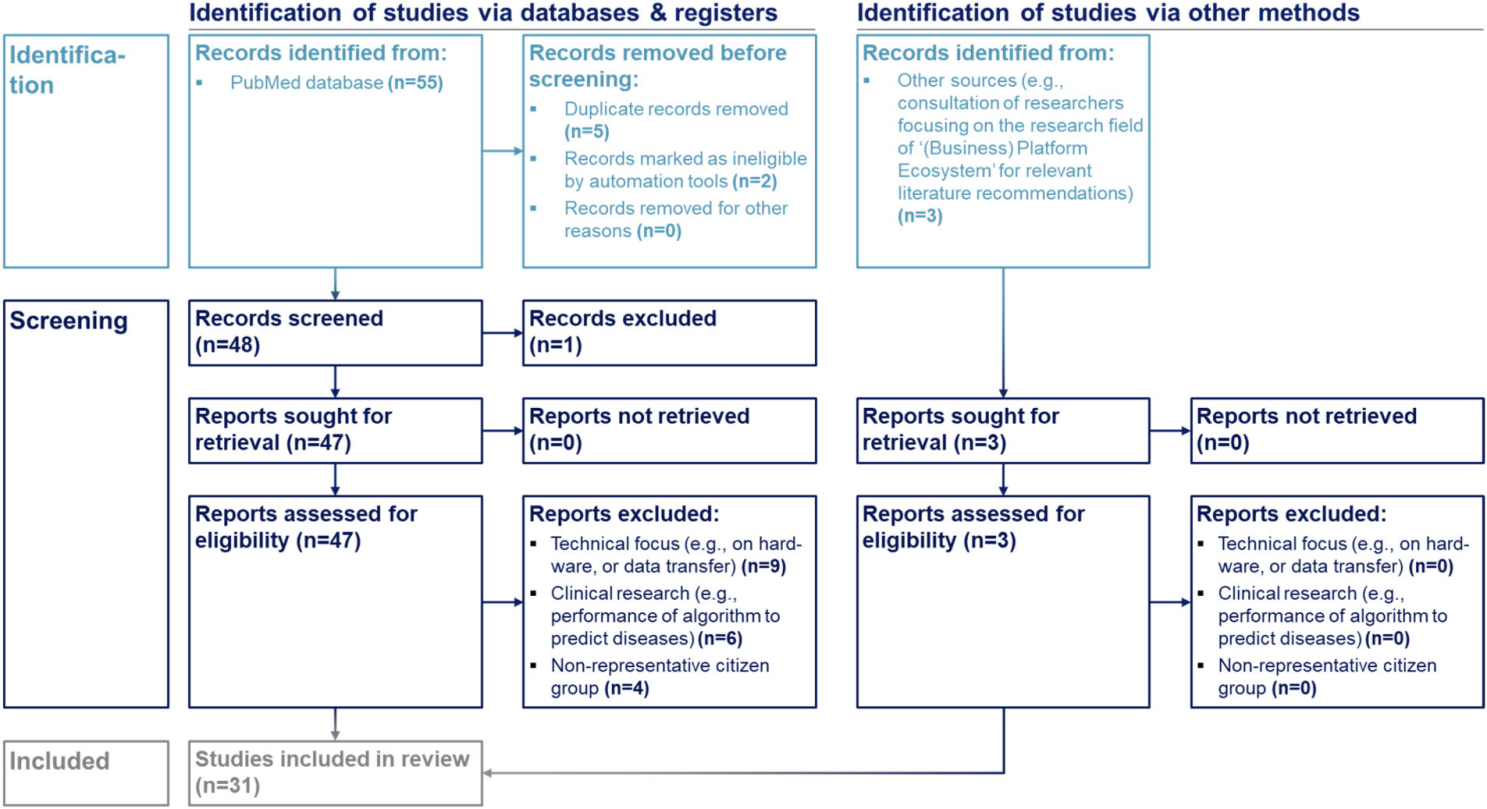
Included studies. Identification and screening process to include relevant literature.

The literature review was guided by the steps of the Preferred Reporting Items for Systematic reviews and Meta-Analyses (PRISMA)-approach for scoping reviews (PRISMA-ScR),^54,55^ as outlined in Figure 1.

The database ‘PubMed’ was used as the source engine and searched based on the following strategy:

[MeSH Terms] ‘(ecosystem) AND (health personnel) AND (telemedicine)’

[MeSH Terms] ‘(ecosystem) AND (humans) AND (telemedicine)’

[Title/Abstract Keyword] ‘(digital health ecosystem) AND (stakeholder)’

The Medical Subject Headings (MeSH) term and keyword selection was inspired by previous research studies using a PRISMA-ScR as standalone methodology with a research question linked to this research study.^9,56,57^ Identification criteria included ‘English language’ and ‘human species’ as filters set in the database automation tool. Duplicates were removed. The title and abstract of the records were screened by two co-authors and excluded if the records were incorrectly classified as eligible by the database's automation filter tool. The remaining records were assessed in their full length for eligibility and excluded if they had a technical focus (e.g. on hardware or data transfer), belonged to clinical research (e.g. performance of algorithm to predict diseases), or focused on non-representative citizen groups (e.g. professional athletes).

To ensure to not exclude or miss any relevant literature from related research fields, in addition to searching the ‘PubMed’ database, other sources were used. The identified literature underwent the same screening process for inclusion as those identified via ‘PubMed’.

The included studies were analysed in two steps: first, a comprehensive overview of the studies in a tabular format was created along the dimensions authors, journal, year of publication, methodology, regional focus, and health service-organizations in scope (Supplementary Results 1). Second, data from the included studies were synthesized by deriving a priori themes and codes representing the initial taxonomy through two co-authors. One co-author allocated the initial a priori coding to the ‘meta-characteristics’, which were structured along the three research questions, merged a priori codes if they were not distinct, renamed them, and clustered them into themes. A priori codes were assigned for relevant results, respectively findings identified by the included studies. A second co-author reviewed the a priori themes and codes. They were either confirmed or discussed between the two co-authors until both were confident with the initial taxonomy to inform the following Delphi interview process, further developing the initial to the final taxonomy through the collection of primary interview data (CtE).

Details of the literature review are reported along the 27-item ‘PRISMA 2020 Checklist’ in Supplementary Methods 1.^58^

### Primary data from semi-structured qualitative expert interviews conducted in two interview rounds using a modified Delphi approach and analysed thematically

The interview guide was designed based on the research questions with the participation of all authors (no permissions were required) and pre-tested with three potential experts to ensure the questions were understandable and effectively addressed the research study's objectives. The answers from these pre-tests were not included in the research study's findings, and these experts were not part of the final expert sample, meaning they were not re-interviewed. Based on the pre-testing, selected questions were slightly reframed. All interviews followed the same interview guide, which started with a section clarifying general information and definitions. Subsequent sections followed the three research questions and combined five open and three one-to-five-point Likert scale-based questions. The Likert scale questions rated expected value-adds, preferred participation roles, and capability gaps. The full interview guide can be found in Supplementary Methods 2.

Following the two Delphi interview rounds, experts in the second round were presented with the interim results (first-round results updated after each second-round interview) specific to their health service-organization group. Delphi agreement was defined as ‘no changes in coding and rounded ratings compared to the interim results’ leading to the discontinuation of interviewing further experts in that group. This led to varying numbers of experts interviewed across stakeholder groups in the second Delphi round. The agreement definition fulfilled the ‘objective ending conditions’ of the taxonomy development methodology.

The design of the modified Delphi approach used in this study has been guided by the ‘Proposed Reporting Guidelines on Delphi Techniques in the Health Sciences’ 15-item checklist and respects the essential components of a traditional Delphi methodology, including participant anonymity, the use of a standardized interview guide, at least one repetition of interviews, and a clear agreement definition.^52^ Details are reported along this 15-item checklist in Supplementary Methods 3.

To derive the interim results, after each interview, interview data were translated into factually anonymized transcripts.^59^ The thematic analysis of the transcripts followed Braun and Clarke's approach, allowing the generation of qualitative and semi-quantitative insights^53^ as grounded theory.^60^ Interview transcripts were coded by two co-authors, who first familiarized themselves with the transcripts, followed by three rounds of coding using MAXQDA (Version 2022.4). First, statements, if classified as relevant by the experts, were either assigned to one of the a priori codes of the initial taxonomy or supplemented as distinct new codes. Second, themes and codes were revised. Third, a second co-author reviewed the coding of the first co-author as coder. Themes and codes were either confirmed or discussed until both were confident that the coding is a fair and adequate representation of the interview data, satisfying the ‘subjective ending conditions’ of the taxonomy development. Arithmetic mean ratings (*M*) and distance between the m ratings of two health service-organization groups (MD) were used for semi-quantitative analyses.

Initial contact with participants was established through written communication, which included information about the research questions, methods, and design. Experts participated voluntarily, had the opportunity to terminate the interview at any time, and were not offered any incentives for participation. Prior to data collection, written informed consent regarding participation, interview audio recording, and publication in a peer-reviewed journal was obtained from all individual experts participating in the study. Further details, including expert recruitment and selection, are reported along the ‘Consolidated Criteria for Reporting Qualitative Research’ (COREQ)^61^ 32-item checklist in Supplementary Methods 4.

## Results

### Literature review

A sample size (*n*) of 31 studies was included, following the identification and screening criteria (Figure 1), conducted on 16 March 2023. A comprehensive overview of the studies included in the literature review is presented in Supplementary Results 1 and confirms the lack of a differentiated understanding of the health service-organization perspective: among the included studies, only four differentiate between two distinct health service-organization groups, and five distinguished more than two. Thereof, only two included studies exclusively focused on health service-organizations. In total, six included studies exclusively focused on health service-organizations, thereof four included providers as the only health service-organization group. Only one study involved German health service-organizations as experts, alongside experts from other European countries, with a total of five out of 22 experts being German. Hence, this study can be considered the first to derive findings from a multi-health service-organization perspective, while distinguishing between distinct groups and reflecting on Germany's dual health system (Supplementary Results 1).

Drawing from the included studies, a priori codes were derived to synthesize and aggregate relevant results and findings of existing literature linked to the research questions. These a priori codes further clustered into themes represented the initial taxonomy. In total, 33 a priori codes were derived at the most granular third coding level. The first coding level was structured along the three research questions, representing the ‘meta-characteristics’ of the taxonomy.

### Semi-structured qualitative expert interviews

The used modified Delphi approach consisted of two rounds of interviews. The first Delphi round took place between 24 April and 12 May 2023, followed by a phase for deriving interim results, and the second Delphi round between 12 June and 7 July 2023.

A total of 21 experts were interviewed. In the first Delphi round, 12 experts were interviewed, equally distributed among the defined four health service-organization groups. In the second Delphi round, an additional nine experts were interviewed, consisting of *n* = 1 payer, *n* = 1 insurer, *n* = 4 providers, and *n* = 3 innovators. A larger *n* of providers and innovators in the second Delphi round was necessary until the Delphi agreement definition was satisfied, given experts’ perspectives within these two groups were less consistent than for insurers and payers. The perspectives across providers tend to be nuanced based on factors such as their affiliation (e.g. university, private, or chains) and position (e.g. chief physician, clinic management, or staff). Perspectives across innovators were nuanced given their different target groups and services (e.g. appointment booking versus online health service). Details about experts’ characteristics are reported in Supplementary Results 2.

The findings from the interviews led to the revision (detailing, renaming, or merging) of the initial 33 a priori codes of the initial taxonomy into 20 codes (CtE). Additionally, 11 new codes (EtC) were supplemented, resulting in a final set of 31 defined codes on the most granular fourth coding level, representing the final taxonomy. Thus, the coding tree of the final taxonomy includes an additional level of detail compared to the initial taxonomy, allowing for a more accurate differentiation of themes and codes. ‘Meta-characteristics’ remained unchanged. The final taxonomy, including the coding tree, code description, and code origination (CtE versus EtC), is presented in Supplementary Results 3. The results of the rating assessment differentiated by the two Delphi interview rounds and four health service-organization groups are presented in Supplementary Results 4. Ratings were conducted using a one-to-five-point Likert scale (one being most, and five being least relevant/fulfilled) and analysed based on the arithmetic mean ratings (*M*) per health service-organization group.

### Expected value-adds and preferred participation roles

**Figure 2.**
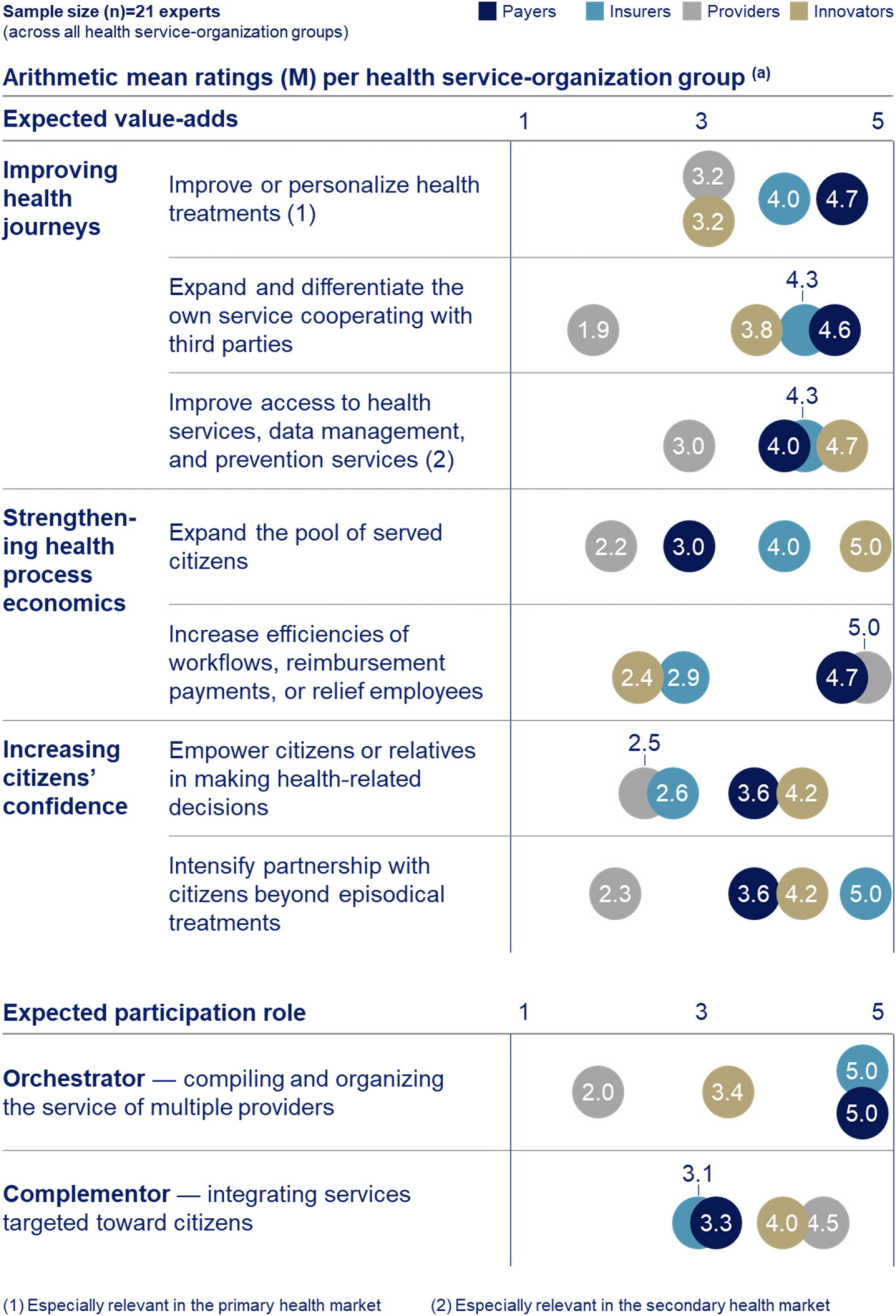
Taxonomy and ratings of expected value-adds from digital health ecosystem participation and preferred participation roles by health service-organization groups. (a) The 21 interviewed experts were asked to rate on a one-to-five-point Likert scale, ranging from 5 (most relevant) to 1 (least relevant). The arithmetic mean ratings (*M*) were calculated by summing the ratings of each expert within a health service-organization group and dividing by the sample size (*n*) of experts interviewed in that group. Developed through a three-step taxonomy development methodology that combines results from a literature review with primary data collected through 21 semi-structured qualitative expert interviews. The interviews were conducted by a modified Delphi approach, consisting of two interview rounds, and the resulting interview data were analysed through thematic analyses. If the *M* values of two health service-organization groups are similar, they are closely aligned regarding the corresponding topic. For expected value-adds being closely aligned suggests a high potential to cooperate. For preferred roles, if two health service-organization groups (like payers and insurer) prefer the ‘orchestrator’ role, they may not match as cooperation partners, given a digital health ecosystem is typically coordinated by one ‘orchestrator’ only. This does not apply to the ‘complementor’ role, as a digital health ecosystem typically integrates the services of multiple health service-organizations as ‘complementors’.

Expected value-adds are clustered in three themes: ‘improving health journeys’, ‘strengthening health process economics’, and ‘increasing citizens’ confidence’, with a total of seven underlying codes. Figure 2 presents the developed taxonomy and ratings differentiated by health service-organization groups.

Providers show a rather opportunistic digital health ecosystem attitude compared to others, as they expect to extract the least value-add (Figure 2). The main overarching reason stated for this is the perceived variability in their day-to-day work, which poses challenges in effectively integrating digital health ecosystems across their workflows:

[Provider] The heterogeneity of patients and variability of the daily clinical work make it almost impossible to integrate digital health ecosystems consistently and efficiently across workflows.

Within the theme ‘improving health journeys’, innovators and insurers aim to ‘improve access to health services, data management, and prevention services’ (*M* = 4.7; 4.3; payers 4.0) which is associated with the secondary health market. Payers focus more on the primary health market to ‘improve or personalize health treatments’ (*M* = 4.7; innovators 3.2; insurers 4.0). Providers rate the latter value-add as the second most relevant (*M* = 3.2) but with distance. Innovators, insurers, and payers anticipate a value-add from ‘expanding and differentiating their own service cooperating with third parties’ (*M* = 3.8; 4.3; 4.6; providers 1.9) (Figure 2).

In terms of ‘strengthening health process economics’, innovators and insurers prioritize revenue growth as they aim to ‘expand the pool of served citizens’ (*M* = 5.0; 4.0; payers 3.0). Providers (*M* = 2.2) do not consider growth opportunities as a value-add (Figure 2) due to the natural high demand they experience:

[Provider] Growth is irrelevant, because we have more than sufficient patients we need to serve today, while that number will naturally grow given the increasing health supply gap.

Compared to innovators and insurers, payers prioritize cost reductions as they aim to ‘increase efficiencies of workflows, reimbursement payments, or relieve employees’ (*M* = 4.7; innovators 2.4; insurers 2.9). Providers also anticipate deriving the main value-add from efficiency gains (*M* = 5.0) (Figure 2). The primary reason stated is the pressure to solve short-term information technology (IT) and workflow efficiency challenges, while their digital health ecosystem thinking is limited mainly to electronic health record (EHR) applications:

[Provider] It would be tremendously beneficial for us to […] know the health history of patients […]. Currently everything is very analog. […] this would reduce workload and costs for example through the avoidance of repeating diagnosis in case of missing results.

Providers’ attribute limited relevance to value-adds in terms of ‘increasing citizens’ confidence’ (Figure 2) as they perceive them to contradict with their main value-add of efficiency gains to strengthening their ‘health process economics’:

[Provider] If you add another digital and open communication channel toward patients, you have the problem, that you will be contacted […] also with challenging questions. […] this would be associated with additional workload, what it should not be […].

In addition to the three roles (‘orchestrator’, ‘complementor’, and ‘enabler’) defined as part of the initial taxonomy, five new sub-themes of these were clustered: (1) ‘Light orchestrator’, which coordinates the service for their clients within a digital health ecosystem organized by another ‘orchestrator’, (2) ‘medical complementor’, which adds a health service, (3) ‘financial complementor’, which adds reimbursement or payment services, (4) ‘regulatory enabler’, which (pseudo-) anonymizes citizen data to make them legally available to other health service-organizations for processing, and (5) ‘technical enabler’, which assists other health service-organizations to address technology and informatic capability gaps (Figure 2) (Supplementary Results 3).

Innovators and providers aim to fulfil the ‘complementor’ role (*M* = 4.0; 4.5). Payers and insurers strive to orchestrate (both *M* = 5.0), while the ‘complementor’ role retains relevance (*M* = 3.3; 3.1) (Figure 2). Results show that providers are not perfectly fit for purpose to orchestrate:

[Provider] Many providers are local constructs and along the health journey only have punctual touchpoints. Making it difficult to build an overarching vision and frequent interactions with citizens […].

### Required capabilities including potential capability gaps

**Figure 3.**
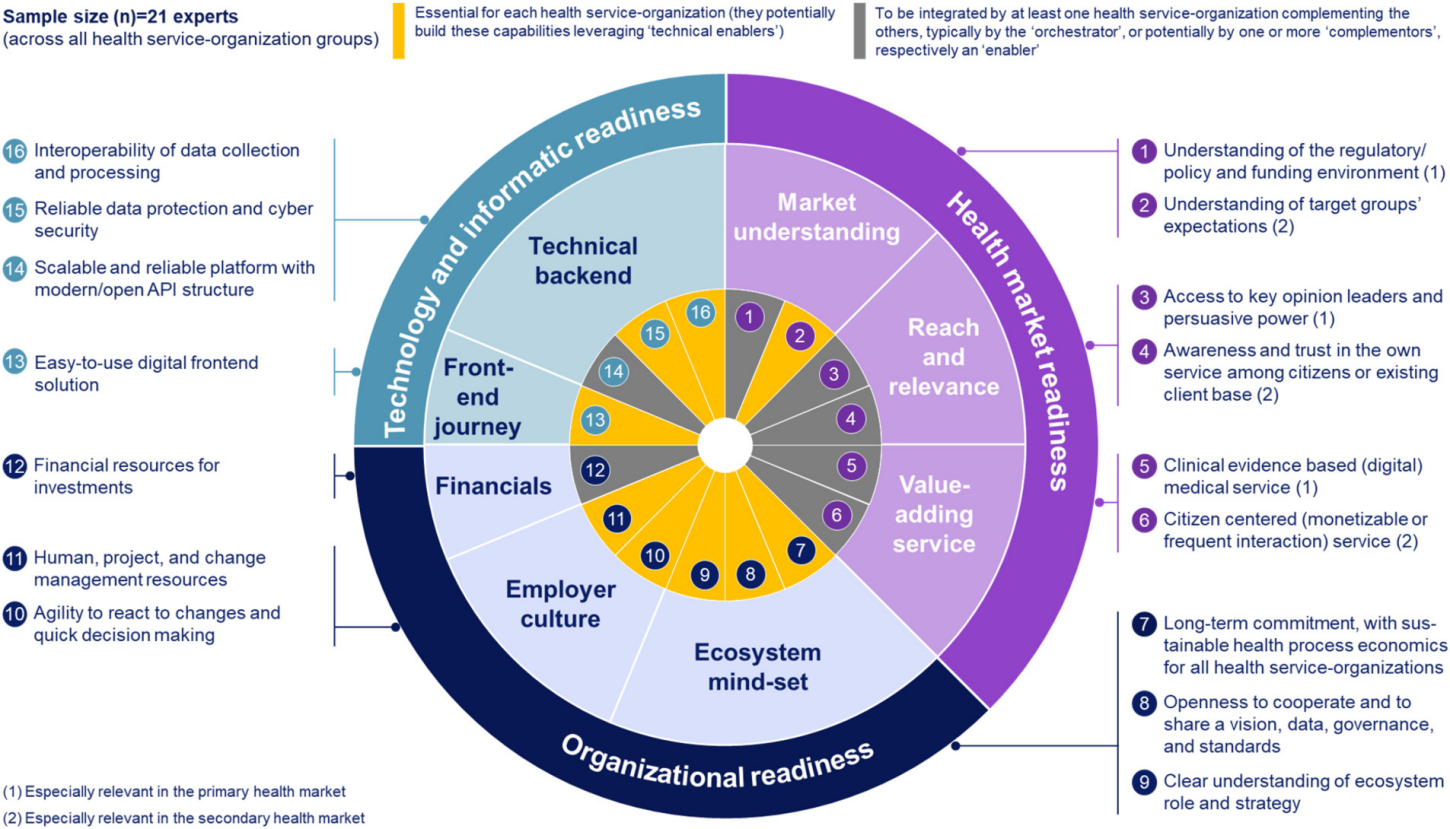
Taxonomy of health service-organizations’ required capabilities to participate in digital health ecosystems. Developed through a three-step taxonomy development methodology that combines results from a literature review with primary data collected through 21 semi-structured qualitative expert interviews. The interviews were conducted by a modified Delphi approach, consisting of two interview rounds, and the resulting interview data were analysed through thematic analyses. The final required capability taxonomy includes 16 capabilities on the most granular fourth coding level. Capabilities highlighted in grey need to be integrated by at least one health service-organization complementing the others, typically by the ‘orchestrator’, or potentially by one or more ‘complementors’, respectively an ‘enabler’. Capabilities highlighted in yellow are essential for each health service-organization to participate in digital health ecosystems (they potentially build these capabilities leveraging ‘technical enablers’).

The required capabilities a priori themes of the initial taxonomy based on Lennon et al.,^25^ were revised, supplemented, and finally categorized along three themes: ‘Health market’, ‘organizational’, and ‘digital’ readiness. These three themes encompass 16 underlying codes, as illustrated in Figure 3, which presents the final developed taxonomy of required capabilities (Supplementary Results 3).

All ‘health market readiness’ capabilities, except for ‘understanding of target groups’ expectations’, might be integrated by a single health service-organization complementing the others. The relevance of capabilities within this theme are nuanced depending on whether the primary or secondary health market is being targeted. ‘Organizational readiness’ capabilities are essential for each health service-organization and cannot be complemented by others, except for ‘financial resources for investments’, which is particularly relevant for the ‘orchestrator’ to invest into awareness and the platform. In line, to provide the platform poses the only ‘technology and informatic’ capability required by a single health service-organization, likely the ‘orchestrator’. All others are essential for each health service-organization, potentially complemented through ‘technical enablers’ (Figure 3).

**Figure 4.**
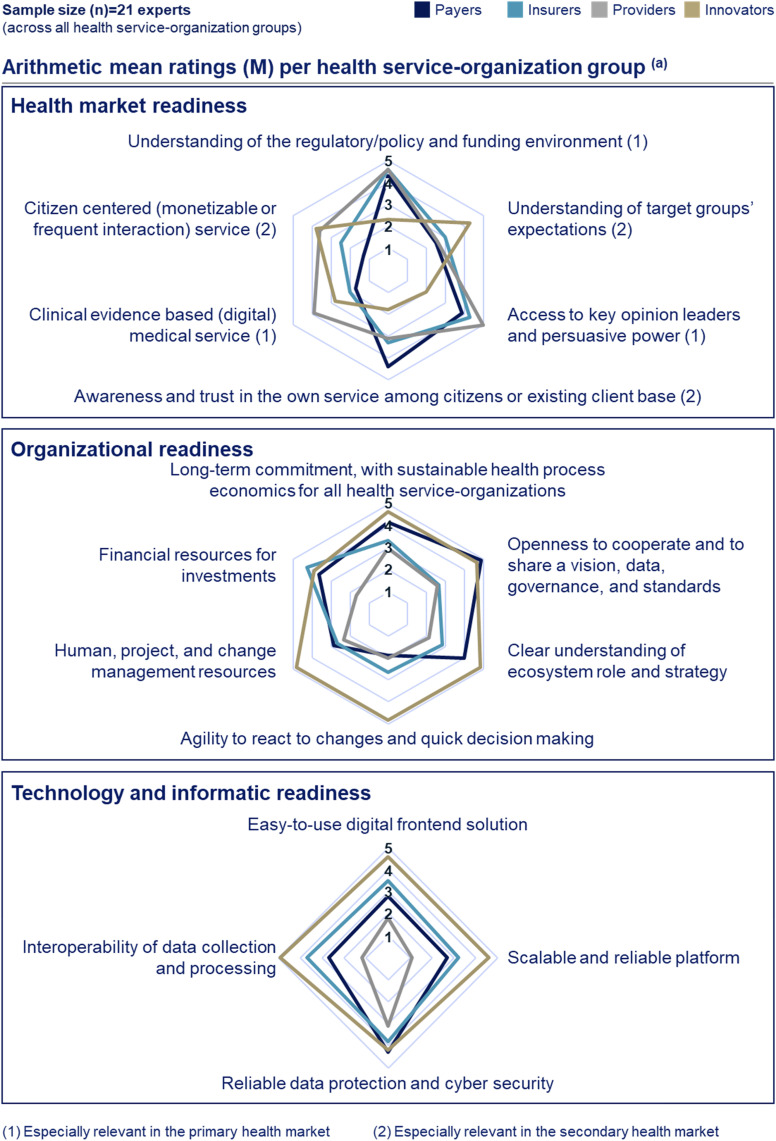
Required capability gap assessment by health service-organization groups. (a) The 21 interviewed experts were asked to rate on a one-to-five-point Likert scale, ranging from 5 (most fulfilled) to 1 (least fulfilled). The arithmetic mean ratings (*M*) were calculated by summing the ratings of each expert within a health service-organization group and dividing by the sample size (*n*) of experts interviewed in that group. Developed through a three-step taxonomy development methodology that combines results from a literature review with primary data collected through 21 semi-structured qualitative expert interviews. The interviews were conducted by a modified Delphi approach, consisting of two interview rounds, and the resulting interview data were analysed through thematic analyses. If the *M* values of two health service-organizations are different for a certain capability, they show potential to complement their capabilities and therewith to cooperate. Only selected capabilities are required from each health service-organization (Figure 3).

Figure 4 presents the results of the capability gap assessment along the defined required capabilities taxonomy. Regarding ‘health market readiness’, payers and innovators show reversed gaps in secondary health market relevant capabilities: payers have the largest capability gaps across others for ‘understanding of target groups’ expectations’ (*M* = 2.5) and ‘citizen centred (monetizable and/or frequent interaction) service’ (*M* = 2.0), which both are strengths of innovators (*M* = 4.3; 3.8). In return, innovators perceive to have the largest capability gap among others in ‘awareness and trust in the own service among citizens or existing client base’ (*M* = 1.8), a strength of payers (*M* = 4.4) (Figure 4).

Providers show the least ‘organizational readiness’ (Figure 4). Results indicate reasons such as different stakeholders with diverging interests (e.g. practitioners, management, and staff), a lack of management professionalism, and different generations:

[Provider] In a hospital you have a lot of stakeholders with different needs […]. Often responsibilities for medical decisions and hospital's management are held by the same person, leading to lacking professional competencies.

Providers perceive to have the least ‘financial resources for investments’ (*M* = 1.7) and ‘human, project, and change management resources’ (*M* = 2.3). Payers’ and innovators’ capabilities differ the most in ‘agility to react to changes and quick decision making’ (*M* = 1.9; 4.8) (Figure 4) posing a potential cooperation barrier:

[Payer] We are missing the ability for agile reactions […]. Driven through […] our public authority structures, including bureaucratic and complicated committee structures.

Among insurers, payers, and innovators, insurers show the lowest ‘ecosystem mind-set’ capabilities, including a limited reliability in terms of long-term commitment (Figure 4):

[Insurer] […] if the ecosystem does not payoff as intended or if management changes, digital health ecosystems strategies and decisions can be altered, and you may exchange your partners or do it yourself.

Providers have the least and innovators the highest ‘technology and informatic readiness’ (Figure 4).

**Figure 5.**
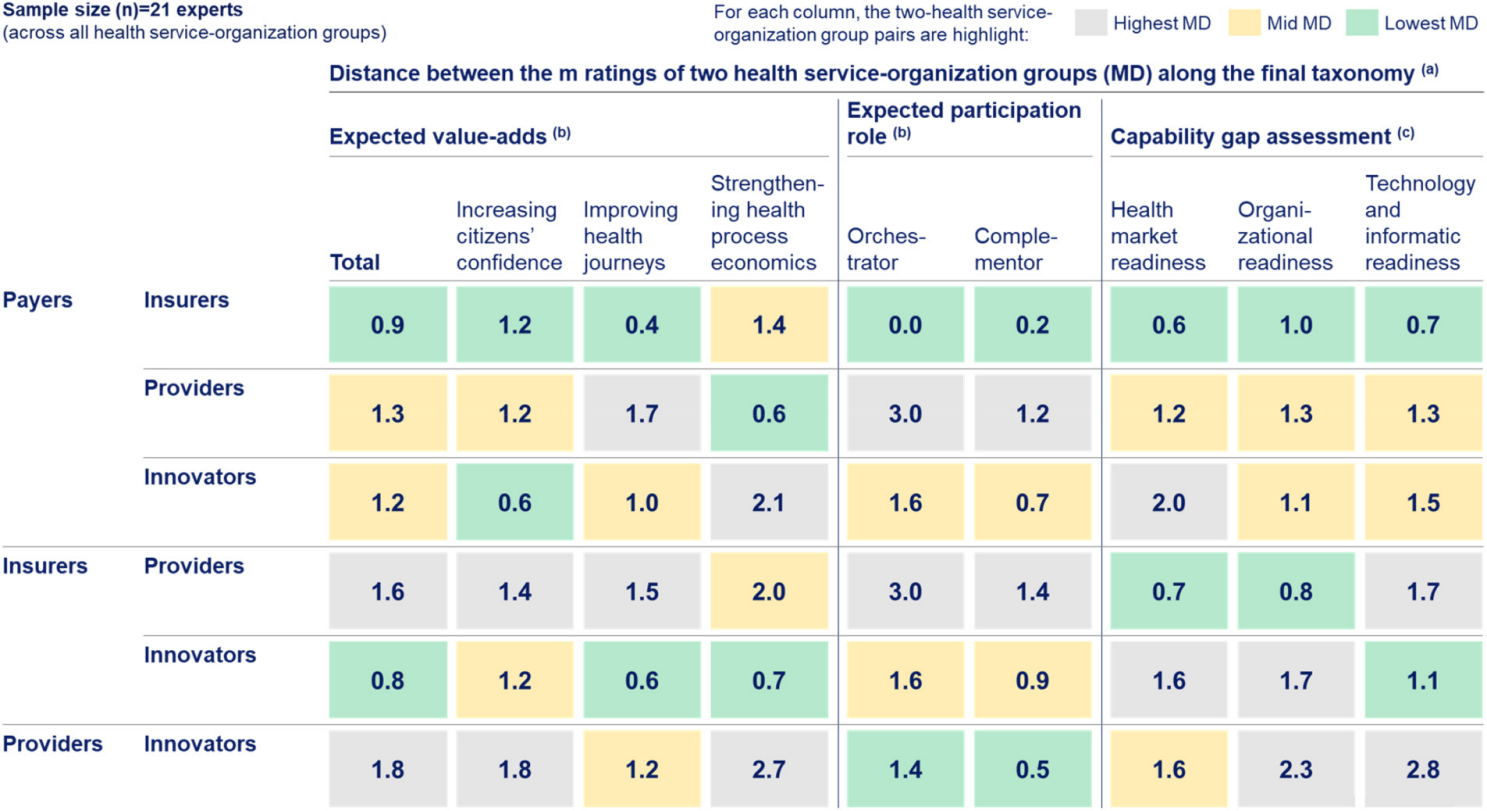
Alignment assessment between health service-organization groups along the final taxonomy. (a) The presented values represent the distance between the *M* ratings of two health service-organization groups (MD). MD is calculated as the *M* of one health service-organization group minus the *M* of another health service-organization group. Given the one-to-five-point Likert scale of the rating assessments, the MD values can range between zero and four. The lower the MD is, the closer aligned two health service-organization groups are. Thus, the cooperation potential of two health service-organization groups increases, if they show a low MD for expected value-adds (being aligned on the value-add to jointly generate), high MD for the ‘orchestrator’ role (as this role is typically taken by one health service-organization only), low MD for the ‘complementor’ role (as a digital health ecosystem typically integrates the service of multiple health service-organizations), and high MD for capability gaps (having different capabilities allowing to complement each other). Each column highlights the two health service-organization group pairs with the lowest MD in green, medium MD in yellow, and highest MD in grey. (b) The underlying one-to-five-point Likert scale ranged from 5 (most relevant) to 1 (least relevant). (c) The underlying one-to-five-point Likert scale ranged from 5 (most fulfilled) to 1 (least fulfilled). Developed through a three-step taxonomy development methodology that combines results from a literature review with primary data collected through 21 semi-structured qualitative expert interviews. The interviews were conducted by a modified Delphi approach, consisting of two interview rounds, and the resulting interview data were analysed through thematic analyses. Providers expected value-adds are the least aligned with other health service-organization. Value-adds anticipated by innovators align more with those of insurers than payers (MD = 0.8; 1.2), primarily driven by differences in ‘strengthening health process economics’ and ‘improving health journeys’. In terms of capability complementation potential, innovators fit better with payers than insurers for ‘health market readiness’ (MD = 2.0; 1.6) and ‘technology and informatic readiness’ (MD = 1.5; 1.1).

Next to the rating assessments for each health service-organization group along the developed taxonomy, this study assessed their alignment by analysing the distance between the *M* ratings of two health service-organization groups (MD) to semi-quantitatively evaluate their cooperation potential, as presented in Figure 5.

## Discussion

### Expected value-adds and preferred participation roles

The ambition of payers and insurers to orchestrate (Figure 2) may be driven by two intentions: first, to address the challenges in the health system, which include the growing health supply gap and rising expenditures,^29–31^ by promoting prevention, access to health services, and remote treatments.^26^ German health service-organization should be particularly concerned about this due to Germany's third-highest health expenditures relative to GDP across OECD countries.^46,47^ Second, the fear of missing out on the potentially economically attractive digital health ecosystem ‘orchestrator’ opportunity or being challenged by new health market players. Thus, some US business professionals, such as Wolf and Pande, believe that the ‘biggest company in the world’^42^ will be a consumer health tech company. Either a payer or insurer emerges toward a ‘payvidor’, owning or providing access to most health services, or a technology native company newly enters the health market, challenging established health players.^42^ This perspective of US business professionals is confirmed as a concern of payers and insurers:

[Insurer] […] in the health system is a huge amount of money and has significant potential […]. Really no one wants to miss this opportunity. Also, some technology companies may have kicked off their activities.

The retained relevance of the ‘complementor’ role for payers and insurers (Figure 2) may be driven by the challenge to build the additional capabilities essential to orchestrate (Figure 3). Particularly for payers, as it entails a shift in their ‘health process economics’ from a focus on costs, encompassing the management of internal costs and primary health market reimbursement payments, to adapting a revenue generation approach. This encompasses integrating secondary health market services such as fostering prevention, data management, and access to health services.^25^ For these services, alternative monetization models are required because the current primary health market reimbursement scheme does not encompass remote treatments and preventive applications. The primary reason for this is insufficient evidence regarding their long-term benefits to the health system.^27^ Similar challenges are observed among US insurers^33^:

[Insurer] […] to bring more services to our existing clients but also to address new ones to grow. It's an investment case, which will or must payoff through additional revenues also beyond today's reimbursement models rather than cost savings.

The revenue focus may also drive innovators’ preference for the ‘complementor’ role (Figure 2) as it allows us to monetize their services quicker, meeting the demands of investors. This might even suggest entering the health market directly rather than spending time integrating into existing solutions at all, respectively to integrate third-parties into the own service,^13^ and at the same time would address the often naturally limited awareness and trust for their services at the beginning.^39^

Providers’ opportunistic attitude aligns with the findings of previous research which indicated a limited citizen-centred perspective of providers,^13^ a limited intention to integrate value-adding services,^33^ and the need of providers to address data flow constraints first.^62^

### Required health market readiness capabilities

The monetization models of digital services are often linked to advertisements or personal data processing but receive limited acceptance in a health-related context by citizens.^39^ Therefore, innovators face a trade-off between focusing their ‘value-adding service’ on the primary versus secondary health market, balancing monetization, and market access considerations. The primary health market is attractive due to services being largely covered by mandatory health insurance. This drives the primary health market's clear reimbursement scheme, relatively large size, consistent standards, and rather homogeneous health insurance coverage among citizens,^63–65^ enabling a clear monetization model to meet the demands of investors.^39^ Access challenges occur from the complex market access approval process, including the required medical studies and regulatory hurdles. Approvals are often restricted to individual nations limiting the scalability. Even Europe as a union lag in creating a one-off across legislation regulatory market access approval.^12^ In addition, the lack of patent protections discourages investing in the market access approval process due to the risk of followers:

[Innovator] The clinical validation is the challenge. It takes time, is costly, and brings uncertainties, while not getting rewarded by a patent protection as for pharmaceuticals. This could lead to followers copying the business model […].

Many innovators already focus on less regulated health promotion, prevention, and overall health-improving services^39^ to avoid market access approval hurdles and therefore do not necessarily deal with regulations first, as described by Lyles et al.^33^ To monetize services outside the reimbursement schemes in the secondary health market, innovators potentially cooperate with insurers, whose clients might be more likely to pay out-of-pocket or insurers themselves may offer partial or full reimbursement for the use of these services in their less regulated environment.^26,40^

The existence of mandatory health insurance, increasing the economic attractiveness of the primary health market, is broadly consistent with other European countries (e.g. UK, France, Sweden, among others) and beyond (e.g. Australia, Singapore, among others). While in most of these countries, mandatory health insurance is mainly publicly sponsored, in the German health system, citizens can choose between the public health market (payers) and the private health market (insurers). This public versus private health market duality may especially impact health service-organizations’ focus to maximize monetization in countries without mandatory health insurance. In these countries, citizens typically acquire their health insurance via the private health markets (e.g. self, or (partially) employer-sponsored), and publicly sponsored health insurance is only offered for certain citizen groups. These citizen groups often include the elderly and underserved. They often account for the highest share of costs in health systems, irrespective of the primary or secondary health market. Thus, representing the target group where most of the value can be monetized.^63,64^ For example, in the USA, publicly sponsored health insurance is provided for elderly (Medicare) and underserved (Medicaid) citizens. For health service-organizations, focusing on the private health market is attractive due to its high price economics. But it comes with low volume and higher heterogeneity, especially regarding the level of health insurance coverage among citizens.^63^ In the public health market economics are driven by high volume but low prices and challenges in reaching the target group,^63^ as they often lack the required enabling technologies,^5,24^ tech savviness,^25,66^ and have diverse needs. This may require the capabilities to combine health-related services with social services^33^ and to reach this target group. The typically decreasing marginal costs of digital services may incentivize health service-organizations to target the high-volume public health market, maximizing monetization.^67^ However, improving innovators’ ‘health process economics’ to address the public health market and attract their investments may require governmental policymakers’ interventions. This could avoid a privatization of digital health, potentially leading to winner-takes-all market dynamics, as observed in private business ecosystems.^68^ Such dynamics could result in a concentration of health supply, that disadvantages certain societal groups and potentially excludes patients with greater needs from having fair access.^5,33,39^ This confirms the ‘inverse care law’, suggesting that access to high-quality health-related services is inversely proportional to the degree of need among citizens.^69^

German payers’ capability gap in ‘understanding of target groups’ expectations’ (Figure 4) may originate from payers’ social purpose to address all citizens, leading to less emphasis on differentiating between various target groups. Therefore, addressing this capability gap would necessitate a shift in German payers’ current approach and focus. Given their higher interactions with elderly citizens, they may lack an understanding of younger target groups with a higher adoption likelihood of digital health ecosystem solutions.^12^ Research showed that ‘one-fits-all-citizens’ digital health ecosystems have limited adaptation potential,^25^ requiring payers to close this capability gap and to differentiate the service toward target groups, potentially through cooperating with innovators.

### Required organizational readiness capabilities

The ‘organizational’ cooperation barrier between payers and innovators (Figure 5) might not only be driven by payers’ public authority structures but also by innovators’ less risk-averse behaviour. For example, ‘hybrid and pragmatic trial-and-error implementation approaches’, as suggested by Spadaro et al. for innovators,^12^ might not be appropriate in the regulated and trust-driven (primary) health market.^70^

The presented findings confirm those of previous studies regarding providers’ limited ‘financial resources for investments’ (Figure 4) being driven by short-term investment priorities to maintain legacy technology and informatic infrastructures as well as to save costs.^27^ Providers’ low ‘openness to cooperate and to share a vision, data, governance, and standards’ (Figure 4) is influenced by their incentive to retain intellectual property for their own publications.^25^ These findings challenge the idea of value creation across health service-organizations through the sharing of cost-savings^71^ and propose data sharing as a potential joint value creation driver, enabling providers to realize efficiency gains and enhance data sources for publications. Thus, the importance of implementing an EHR as enabler with common data standards is emphasized. But the benefit of data sharing from a joint data pool may be rather of long-term nature, as it requires time to build up a critical mass of data before value can be extracted. This time lag until the value can be realized may lead health service-organization to deprioritize data integration in the short term. The deferral of gratification could require interventions from governmental policymakers.

The low capabilities in ‘human, project, and change management resources’ among providers (Figure 4) aligns with a survey indicating that the German health system workforce feels least prepared for the digitization in their work environment across sector.^37^ In addition, previous interactions with digitization efforts might be associated with negative experiences, which could potentially demotivate to adapt to digital health ecosystems with even more profound changes.^28^ For example, going forward, the role of providers’ workforce may include educating citizens on the use of digital health technologies and aiding them in distinguishing between valuable and irrelevant services and information.^32^ This suggests for governmental policymakers to act, closing the capability gaps across health service-organizations’ workforces.

### Required technology and informatic readiness capabilities

Payers’ and insurers’ low ‘technology and informatic’ capability (Figure 4) is driven by fragmented health journeys and limiting legacy IT.^19,25^ A solution may be spinning off activities to rebuild IT on a ‘green field’ to reduce dependency from legacy IT, potentially less applicable for payers given their higher regulatory boundaries. Another solution may be leveraging ‘technical enablers’, which has grown in attractiveness given their increased maturity.^66,72^

Despite providers’ limited expected value-adds (Figure 2) and capability fulfilment (Figure 4), their integration into digital health ecosystems is essential due to their central position in the health network, referring to their medical service^40^:

[Provider] […] we offer a core service. […] We have natural interactions like no one else. Citizens contact us in a pull-principal. […] Even if interaction frequencies might be low during some periods, we are an anchor that lets citizens return, as we are unreplaceable in a health journey.

This suggests the need to encourage providers to participate, including emphasizing efficiency potentials through the sharing of patient data and service training to enable employees.^12,24,27,28,32,40^ Incentives or health policy measures might be necessary:

[Innovator] Practitioners need to be convinced from the value-add in terms of quicker workflows and money to earn. Maybe you even need to financially incentivize them to share data. As ultimate solution, the regulator may pay for it or enforce it lawfully.

### Implications for health service-organization groups as part of the expert sample and beyond, governmental policymakers, and citizens

**Figure 6.**
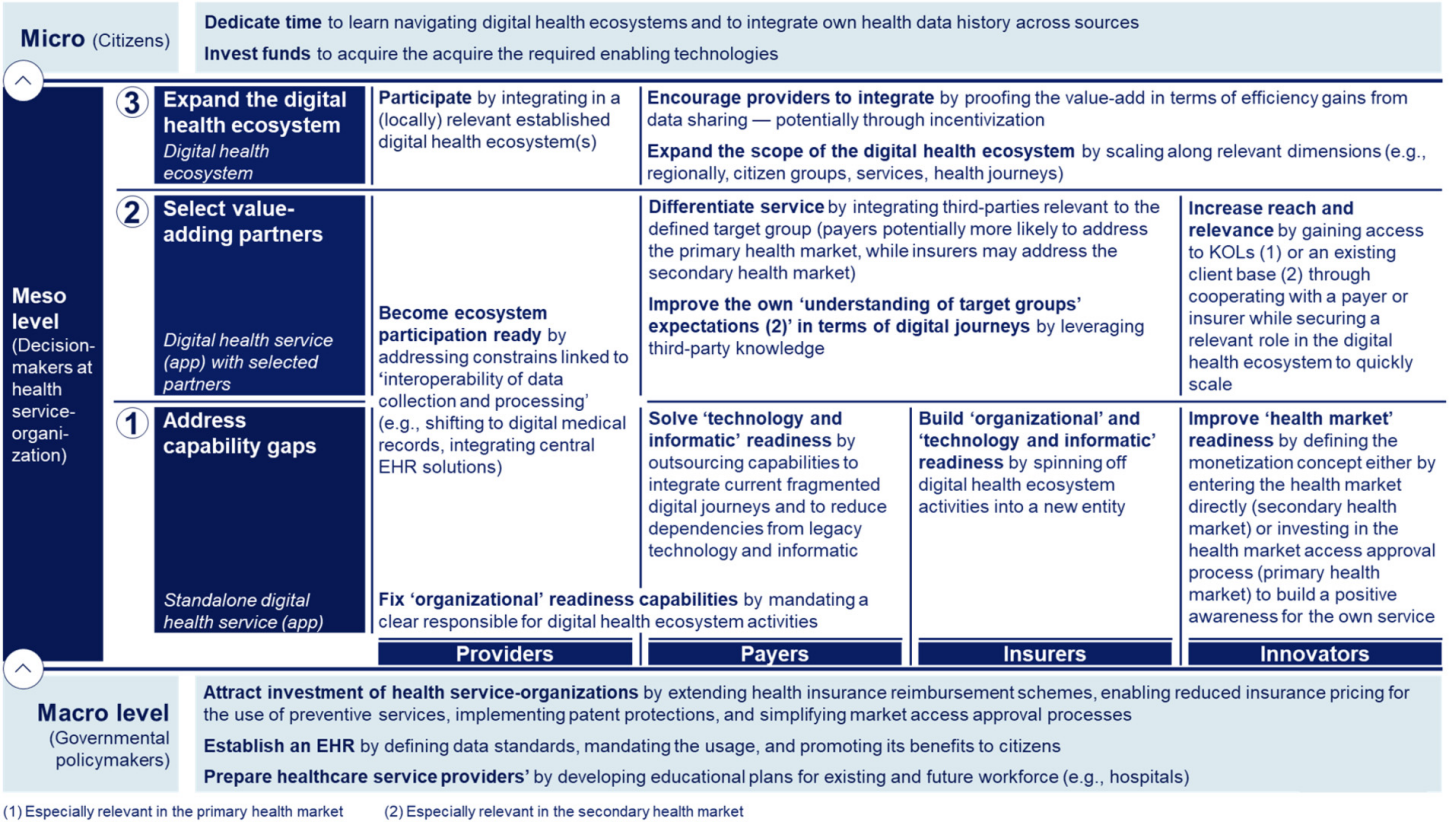
Three-staged approach toward digital health ecosystems. Developed through a three-step taxonomy development methodology, combining results from a literature review with primary data collected through 21 semi-structured qualitative expert interviews. The interviews were conducted by a modified Delphi approach, consisting of two interview rounds, and the resulting interview data were analysed through thematic analyses. Health service-organizations worldwide can assess their starting stage based on a rating assessment along the final developed taxonomy to prioritize their actions accordingly.

The findings suggest sequentially building the required digital health ecosystem capabilities, as outlined in Figure 6. First, health service-organizations need to individually ‘address capability gaps’. Innovators may directly enter the health market to build positive awareness for their services. Second, health service-organizations should ‘select value-adding partners’ to address remaining capability gaps. Innovators require access to key opinion leaders (KOL) or an existing client base to scale. Payers and insurers should integrate differentiating third-party services. While providers might need more time to ‘address capability gaps’, in the third stage they should be integrated given their essential position to ‘expand toward a digital health ecosystem’, potentially encouraged through incentives (Figure 6).

Health service-organizations’ groups that have not been interviewed as part of the expert sample (e.g. ‘Big Techs’, hospital-information-system companies) may still extract value from these findings. The required capabilities (Figure 3) (Supplementary Results 3) are applicable across all health service-organization groups and hold relevance for both offering a standalone digital health-related service and participating within digital health ecosystems. ‘Technology and informatic’ and ‘health market’ capabilities are required to offer a digital health app tailored to the distinct health market characteristics. ‘Organizational’ capabilities are required, when participating in a digital health ecosystem. Additionally, all health service-organization groups can identify capabilities to invest in, carving out a distinctive value they could complement a digital health ecosystem with. Thereby, taking on the role of closing the required capability gaps of others, becoming a relevant partner. In return, own required capability gaps may be addressed through others. For example, ‘Big Techs’ such as Apple or hospital-information-system companies may face gaps related to the capability ‘awareness and trust in the own service among citizens or existing client base’ within the German health market. They could use the findings to identify suitable partners to bridge this gap, while bringing in their potential strength of a ‘scalable and reliable platform with modern/open API structure’, addressing a capability gap for most health service-organizations (except for innovators) (Figure 4).

The discussion reveals the importance of governmental policymakers and citizens in closing health-service organizations’ required capability gaps, as well as the supply and demand gap, to establish digital health ecosystems. Health service-organization may even rely on them to some extent. Governmental policymakers need to implement standards and policies aimed at enhancing innovators’ ‘health process economics’ to attract private investments, given their significant role in driving health innovations.^39^ Hence, governmental policyholders could expand reimbursement schemes to include secondary health market services, allowing payers to adjust insurance pricing for incentives toward preventive services. However, as the primary health market is 2.8 times the size of the secondary health market, private investments in this market could be incentivized.^65^ Potential actions include simplifying the primary health market approval processes (e.g. faster processes for health-related services addressing less critical topics) combined with patent protection for innovative digital health-related services, which may be at risk of being copied by competitors. In countries with a private versus public health market duality, governmental policymakers should additionally improve the economic attractiveness for private health service-organizations to operate in the public health market by targeting elderly and underserved citizens, thereby transforming the health system where most of its costs occur. Possibilities include further regulatory market opening, establishing more market standards, and equipping citizens with enabling technologies (e.g. smartphones, wearables), all to simplify market access and to leverage benefits from high-volume economics. Additionally, providing financial subsidies could improve pricing economics.

In addition, governmental policymakers may need to intervene to address the low EHR adoptions. This includes defining common data standards, as well as solving the time lag between entering data and extracting gratification from a joint data pool for health service-organizations. Possibly through financial incentives for EHR adoption and data integration, potentially even making usage mandatory for payers and public providers.^73^ The need to act was acknowledged by the German governmental policymaker. Payers are now regulatory enforced to offer the EHR usage to citizens and regulations transitioned from an ‘opt-in’ to an ‘opt-out’ approach for citizens regarding their EHR usage starting in 2025.^74^ Beyond, policymakers could actively promote the existence and benefits of the EHR to citizens, as adoption rates remain low.^75^

The findings also suggest that providers, payers, and insurers may rely on governmental policymakers’ support to address the capability gap concerning their workforce. Thus, governmental policymakers should adjust the curricula of training programs (including academic education) to prepare the future workforce and to enable the existing one.^32,76^

Citizens may also need to increase their time investment in learning how to navigate digital health ecosystems to experience the benefits and in integrating their health history comprehensively, creating an increased demand for the EHR. Additionally, they may need to invest in enabling technologies such as smartphones and wearables, especially pronounced for certain societal groups or in less developed countries^77,78^ (Figure 6).

## Conclusions

### Three-staged approach toward digital health ecosystems

The findings lead to actionable recommendations for health service-organizations structured in a three-staged approach. Stages one and two focus on establishing a digital service within the health market, building the prerequisite to expand toward a digital health ecosystem offering in the third stage. Health service-organizations worldwide can determine their starting stage based on an individual capability assessment. Health service-organization groups not part of the expert sample (e.g. ‘Big Techs’, hospital-information-system companies) can utilize the three-staged approach for either building the required capabilities to offer a standalone digital health-related service or identifying capabilities where they can complement value for ecosystem partners.

The discussion revealed that both governmental policymakers and citizens also play a role in closing required capability gaps of health service-organizations for establishing digital health ecosystems.

Governmental policymakers should prioritize three health policy initiatives: first, policies that attract investments in digital health-related services by extending health insurance reimbursement schemes for digital-related health services, implementing patent protections, and simplifying market access approval processes. Countries which require a higher engagement of health-service organizations in the public health market should consider, in addition, to open-up regulations, establish more market standards, equip citizens with enabling technologies (e.g. smartphones or wearables), and subsidize prices. Second, the implementation of an EHR through common data standards, potentially a regulatorily mandated or incentivized use by payers and providers, as well as promoting its benefits to citizens. Third, initiatives to prepare healthcare service-providers’ workforce.

Last, citizens are required to take a more active role by investing in enabling technologies, learning to navigate digital health ecosystems, ‘opting-in’ to relevant enablers like the EHR, and integrating personal health data.

### Strengths and limitations

To the best of the author's knowledge, the findings of this study make a distinctive contribution as they are based on the first primary data collection that encompasses a comprehensive multi-health service-organization perspective. Thus, the findings contribute to setting the foundations for further research, while providing actionable recommendations that aid health service-organizations in prioritizing actions to establish digital health ecosystems. With the sample focused on Germany's dual health system, which covers the common structures of health systems worldwide, the relevance of these findings extends to health service-organizations globally.

This study comes with three limitations, while most are typically for the used interview methodology: first, results might be influenced by the experiences of the participating experts. The defined stakeholder groups themselves could be further differentiated into ‘sub-groups’ based on their backgrounds, driving heterogenous experiences. This differentiation could either be based on experts’ positions, distinguishing between those with direct profit and loss responsibility (e.g. management) versus a functional role (e.g. physician in a hospital, app developer), or the organizational type they work for (e.g. public sponsored, for-profit). Second, a bias might arise from a self-selection of participating experts who were more positively inclined toward the research questions. Mitigation measures included a careful definition of criteria to define experts, the formation of groups, and defining an objective Delphi agreement definition to determine content saturation, which led to the discontinuation of interviews. Third, despite the multi-health service-organization perspective, the findings are derived from a focused sample at the meso level. Perspectives of some health service-organization groups (e.g. hospital-information-system companies, or ‘Social Media’ and ‘Big Tech’ companies such as Apple), stakeholders at the macro level (governmental policymakers), and micro level (citizens) were not investigated through primary data from interviews.

Beyond limitations associated with the interviews, health service-organizations’ priorities may vary depending on the health system structure in which they operate. Variances may occur due to differences in the availability of mandatory insurance and the share of private insurers’ (private health market) and versus payers’, respectively governmental policymakers sponsored insurances (public health market).

### Suggestions for further research enquiries

Based on the findings of this study, four directions for further scientific enquiry emerge: first, the research questions could be further investigated for other stakeholder groups at the meso level, particularly ‘Social Media’ and ‘Big Tech’ companies.

Second, research should further concretize the initiatives to be prioritized by governmental policymakers, as well as the attitudes of citizens. Thus, the roles of stakeholders beyond health service-organizations (at the micro and macro levels) in supporting the establishment of digital health ecosystems and addressing the required capability gaps of health service-organizations should be detailed.

Third, further research could investigate whether market concentration dynamics would hold true for health systems and assess its implications for a fair access across societal groups.

Fourth, considering the developing research field, further research could contribute by integrating results across research studies to align the understanding of digital health ecosystems. This could be achieved through a scoping review as a standalone methodology, using an updated and broadened set of MeSH terms.

## Supplemental Material

sj-docx-1-dhj-10.1177_20552076241271890 - Supplemental material for How to establish digital health ecosystems from the perspective of health service-organizations: A taxonomy developed based on expert interviews conducted as modified Delphi approach Supplemental material, sj-docx-1-dhj-10.1177_20552076241271890 for How to establish digital health ecosystems from the perspective of health service-organizations: A taxonomy developed based on expert interviews conducted as modified Delphi approach by Robin Huettemann, Benedict Sevov, Sven Meister and Leonard Fehring in DIGITAL HEALTH

sj-docx-2-dhj-10.1177_20552076241271890 - Supplemental material for How to establish digital health ecosystems from the perspective of health service-organizations: A taxonomy developed based on expert interviews conducted as modified Delphi approach Supplemental material, sj-docx-2-dhj-10.1177_20552076241271890 for How to establish digital health ecosystems from the perspective of health service-organizations: A taxonomy developed based on expert interviews conducted as modified Delphi approach by Robin Huettemann, Benedict Sevov, Sven Meister and Leonard Fehring in DIGITAL HEALTH

sj-docx-3-dhj-10.1177_20552076241271890 - Supplemental material for How to establish digital health ecosystems from the perspective of health service-organizations: A taxonomy developed based on expert interviews conducted as modified Delphi approach Supplemental material, sj-docx-3-dhj-10.1177_20552076241271890 for How to establish digital health ecosystems from the perspective of health service-organizations: A taxonomy developed based on expert interviews conducted as modified Delphi approach by Robin Huettemann, Benedict Sevov, Sven Meister and Leonard Fehring in DIGITAL HEALTH

sj-docx-4-dhj-10.1177_20552076241271890 - Supplemental material for How to establish digital health ecosystems from the perspective of health service-organizations: A taxonomy developed based on expert interviews conducted as modified Delphi approach Supplemental material, sj-docx-4-dhj-10.1177_20552076241271890 for How to establish digital health ecosystems from the perspective of health service-organizations: A taxonomy developed based on expert interviews conducted as modified Delphi approach by Robin Huettemann, Benedict Sevov, Sven Meister and Leonard Fehring in DIGITAL HEALTH

sj-docx-5-dhj-10.1177_20552076241271890 - Supplemental material for How to establish digital health ecosystems from the perspective of health service-organizations: A taxonomy developed based on expert interviews conducted as modified Delphi approach Supplemental material, sj-docx-5-dhj-10.1177_20552076241271890 for How to establish digital health ecosystems from the perspective of health service-organizations: A taxonomy developed based on expert interviews conducted as modified Delphi approach by Robin Huettemann, Benedict Sevov, Sven Meister and Leonard Fehring in DIGITAL HEALTH

sj-docx-6-dhj-10.1177_20552076241271890 - Supplemental material for How to establish digital health ecosystems from the perspective of health service-organizations: A taxonomy developed based on expert interviews conducted as modified Delphi approach Supplemental material, sj-docx-6-dhj-10.1177_20552076241271890 for How to establish digital health ecosystems from the perspective of health service-organizations: A taxonomy developed based on expert interviews conducted as modified Delphi approach by Robin Huettemann, Benedict Sevov, Sven Meister and Leonard Fehring in DIGITAL HEALTH

sj-docx-7-dhj-10.1177_20552076241271890 - Supplemental material for How to establish digital health ecosystems from the perspective of health service-organizations: A taxonomy developed based on expert interviews conducted as modified Delphi approach Supplemental material, sj-docx-7-dhj-10.1177_20552076241271890 for How to establish digital health ecosystems from the perspective of health service-organizations: A taxonomy developed based on expert interviews conducted as modified Delphi approach by Robin Huettemann, Benedict Sevov, Sven Meister and Leonard Fehring in DIGITAL HEALTH

sj-docx-8-dhj-10.1177_20552076241271890 - Supplemental material for How to establish digital health ecosystems from the perspective of health service-organizations: A taxonomy developed based on expert interviews conducted as modified Delphi approach Supplemental material, sj-docx-8-dhj-10.1177_20552076241271890 for How to establish digital health ecosystems from the perspective of health service-organizations: A taxonomy developed based on expert interviews conducted as modified Delphi approach by Robin Huettemann, Benedict Sevov, Sven Meister and Leonard Fehring in DIGITAL HEALTH

## References

[1] Neupert P Mundie C . Personal health management systems: applying the full power of software to improve the quality and efficiency of care. Health Aff (Millwood) 2009; 28: 390–392. 19275994 10.1377/hlthaff.28.2.390

[2] Mosa ASM Yoo I Sheets L . A systematic review of healthcare applications for smartphones. BMC Med Inform Decis Mak 2012; 12: 67. 22781312 10.1186/1472-6947-12-67 PMC3534499

[3] Eysenbach G . What is e-health? J Med Internet Res 2001; 3: e20. 10.2196/jmir.3.2.e20 PMC1761894 11720962

[4] Chakraborty I Edirippulige S Vigneswara Ilavarasan P . The role of telehealth startups in healthcare service delivery: a systematic review. Int J Med Inform 2023; 174: 105048. 36963322 10.1016/j.ijmedinf.2023.105048

[5] Vujosevic S Limoli C Luzi L , et al. Digital innovations for retinal care in diabetic retinopathy. Acta Diabetol 2022; 59: 1521–1530. 35962258 10.1007/s00592-022-01941-9 PMC9374293

[6] Burrell A Zrubka Z Champion A , et al. How useful are digital health terms for outcomes research? An ISPOR special interest group report. Value Health 2022; 25: 1469–1479. 36049797 10.1016/j.jval.2022.04.1730

[7] World Health Organization. Global strategy on digital health 2020–2025. Geneva: World Health Organization, 2021.

[8] Baumel A Muench F Edan S , et al. Objective user engagement with mental health apps: systematic search and panel-based usage analysis. J Med Internet Res 2019; 21: e14567. 10.2196/14567 PMC6785720 31573916

[9] Alvandi AO Bain C Burstein F . Understanding digital health ecosystem from Australian citizens’ perspective: a scoping review. PLoS One 2021; 16: e0260058. 10.1371/journal.pone.0260058 PMC8592460 34780547

[10] Goetz M Müller M Matthies LM , et al. Perceptions of patient engagement applications during pregnancy: a qualitative assessment of the patient's perspective. JMIR mHealth uHealth 2017; 5: e73. 10.2196/mhealth.7040 PMC5466700 28550005

[11] Benis A Tamburis O Chronaki C , et al. One digital health: a unified framework for future health ecosystems. J Med Internet Res 2021; 23: e22189. 10.2196/22189 PMC7886486 33492240

[12] Spadaro B Martin-Key NA Bahn S . Building the digital mental health ecosystem: opportunities and challenges for mobile health innovators. J Med Internet Res 2021; 23: e27507. 10.2196/27507 PMC8552100 34643537

[13] Iyawa GE Herselman M Botha A . Digital health innovation ecosystems: from systematic literature review to conceptual framework. Procedia Comput Sci 2016; 100: 244–252.

[14] Avramakis E Anchen J Raverkar AK . Health ecosystems towards an integrated and seamless patient experience: digital ecosystems series. Zürich: SwissRe Institute, 2019.

[15] Raab A Unterbrunner M . Patient experience und patient journey als maßgebliche konzepte für den digitalen wandel im gesundheitswesen. In: Stummeyer C Raab A Behm ME (eds) Plattformökonomie im gesundheitswesen. 1st ed. Wiesbaden: Springer Fachmedien Wiesbaden, 2023, pp.3–14.

[16] Schreiweis B Pobiruchin M Strotbaum V , et al. Barriers and facilitators to the implementation of eHealth services: systematic literature analysis. J Med Internet Res 2019; 21: e14197. 10.2196/14197 PMC6898891 31755869

[17] Fatehi F Samadbeik M Kazemi A . What is digital health? Review of definitions. Stud Health Technol Inform 2020; 275: 67–71. 33227742 10.3233/SHTI200696

[18] Baghbadorani MF Harandi A . A conceptual model for business ecosystem and implications for future research. IPEDR 2012; 52: 82–86.

[19] Marcelino I Laza R Domingues P , et al. Active and assisted living ecosystem for the elderly. Sensors 2018; 18: 1246. 29673234 10.3390/s18041246 PMC5948742

[20] Moore JF . The death of competition: leadership and strategy in the age of business ecosystems. XIII. New York: Harper Business, 1996.

[21] Floch J Zettl A Fricke L , et al. User needs in the development of a health app ecosystem for self-management of cystic fibrosis: user-centered development approach. JMIR mHealth uHealth 2018; 6: e113. 10.2196/mhealth.8236 PMC5964302 29739742

[22] Moulin T Dobson J Hixson JD , et al. Digital health: the journal's pioneering journey 6 years on. Digit Health 2021; 7: 20552076211034034. 34777851 10.1177/20552076211034034 PMC8588798

[23] Parikh RB Basen-Enquist KM Bradley C , et al. Digital health applications in oncology: an opportunity to seize. J Natl Cancer Inst 2022; 114: 1338–1339. 35640986 10.1093/jnci/djac108 PMC9384132

[24] Beneteau E Paradiso A Pratt W . Telehealth experiences of providers and patients who use augmentative and alternative communication. J Am Med Inform Assoc 2022; 29: 481–488. 34897460 10.1093/jamia/ocab273 PMC8800527

[25] Lennon MR Bouamrane M-M Devlin AM , et al. Readiness for delivering digital health at scale: lessons from a longitudinal qualitative evaluation of a national digital health innovation program in the United Kingdom. J Med Internet Res 2017; 19: e42. 10.2196/jmir.6900 PMC5334516 28209558

[26] Thomas EE Haydon HM Mehrotra A , et al. Building on the momentum: sustaining telehealth beyond COVID-19. J Telemed Telecare 2022; 28: 301–308. 32985380 10.1177/1357633X20960638

[27] Diaz-Skeete Y Giggins OM McQuaid D , et al. Enablers and obstacles to implementing remote monitoring technology in cardiac care: a report from an interactive workshop. Health Informatics J 2020; 26: 2280–2288. 31854212 10.1177/1460458219892175

[28] Putteeraj M Bhungee N Somanah J , et al. Assessing E-health adoption readiness using diffusion of innovation theory and the role mediated by each adopter's category in a Mauritian context. Int Health 2022; 14: 236–249. 34114007 10.1093/inthealth/ihab035 PMC9070468

[29] Zaman SB Hossain N Mehta V , et al. An association of total health expenditure with GDP and life expectancy. J Med Res Innov 2017; 1: AU7–AU12.

[30] Deutsche Krankenhaus Gesellschaft. Fallzahlenrückgänge sind alarmierend – DKG befürchtet Versorgungslücken und Langzeitfolgen, https://www.dkgev.de/dkg/presse/details/fallzahlenrueckgaenge-sind-alarmierend-dkg-befuerchtet-versorgungsluecken-und-langzeitfolgen/ (2023, accessed 26 June 2023).

[31] Braeseke G Huster S Pflug C , et al. Studie zum Versorgungsmanagement durch Patientenlotsen, https://www.bundesgesundheitsministerium.de/fileadmin/Dateien/5_Publikationen/Praevention/Berichte/IGES_Versorgungsmanagement_durch_Patientenlotsen_042018.pdf (2018, accessed 26 June 2023).

[32] Győrffy Z Radó N Mesko B . Digitally engaged physicians about the digital health transition. PLoS One 2020; 15: e0238658. 10.1371/journal.pone.0238658 PMC7521720 32986733

[33] Lyles CR Adler-Milstein J Thao C , et al. Alignment of key stakeholders’ priorities for patient-facing tools in digital health: mixed methods study. J Med Internet Res 2021; 23: e24890. 10.2196/24890 PMC8430871 34435966

[34] van Kessel R Roman-Urrestarazu A Anderson M , et al. Mapping factors that affect the uptake of digital therapeutics within health systems: scoping review. J Med Internet Res 2023; 25: e48000. 10.2196/48000 PMC10410406 37490322

[35] Research 2 Guidance. mHealth Economics 2017 – Current Status and Future Trends in Mobile Health, https://research2guidance.com/product/mhealth-economics- (2017).

[36] Borycki EM Kushniruk AW . Reinventing virtual care: bridging the healthcare system and citizen silos to create an integrated future. Healthc Manage Forum 2022; 35: 135–139. 35473445 10.1177/08404704211062575 PMC9047099

[37] Stürz RA Schlude A Putfarken H , et al. Das bidt-SZ Digitalbarometer. Munich: bidt - Bayerisches Forschungsinstitut für Digitale Transformation, 2022.

[38] Biesdorf S Deetjen U Kayyali B . Digital health ecosystems: digital health ecosystems: voices of key healthcare leaders, https://www.mckinsey.com/industries/healthcare/our-insights/digital-health-ecosystems-voices-of-key-healthcare-leaders (2021, accessed 13 February 2023).

[39] Grundy Q . A review of the quality and impact of mobile health apps. Annu Rev Public Health 2022; 43: 117–134. 34910582 10.1146/annurev-publhealth-052020-103738

[40] Park Y Park S Lee M . Digital health care industry ecosystem: network analysis. J Med Internet Res 2022; 24: e37622. 10.2196/37622 PMC9434390 35976690

[41] Dedehayir O Mäkinen SJ Roland Ortt J . Roles during innovation ecosystem genesis: a literature review. Technol Forecast Soc Change 2018; 136: 18–29.

[42] Wolf D Pande V . The Biggest Company in the World, https://a16z.com/2022/11/11/the-biggest-company-in-the-world/ (2022, accessed 6 March 2023).

[43] Hausmann JS Touloumtzis C White MT , et al. Adolescent and young adult use of social media for health and its implications. J Adolesc Health 2017; 60: 714–719. 28259620 10.1016/j.jadohealth.2016.12.025 PMC5441939

[44] Rolnick J Ward R Tait G , et al. Early adopters of Apple health records at a large academic medical center: cross-sectional survey of users. J Med Internet Res 2022; 24: e29367. 10.2196/29367 PMC8826150 35076397

[45] Peeters JM Krijgsman JW Brabers AE , et al. Use and uptake of eHealth in general practice: a cross-sectional survey and focus group study among health care users and general practitioners. JMIR Med Inform 2016; 4: e11. 10.2196/medinform.4515 PMC4838754 27052805

[46] OECD/European Observatory on Health Systems and Policies. Deutschland: Länderprofil Gesundheit 2019: State of Health in the EU. Brussels: OECD Publishing, Paris/European Observatory on Health Systems and Policies, 2019.

[47] Müller M . Das deutsche Gesundheitssystem im internationalen Vergleich, https://blog.oecd-berlin.de/das-deutsche-gesundheitssystem-im-internationalen-vergleich (2020, accessed 7 March 2023).

[48] OECD Economics Department Policy Notes. Health care systems: getting more value for money. 2nd ed. Paris: OECD Publishing, OECD Economics Department Policy Notes, 2010.

[49] Bundesministerium für Gesundheit. Gesundheitswirtschaft im Überblick, https://www.bundesgesundheitsministerium.de/themen/gesundheitswesen/gesundheitswirtschaft/gesundheitswirtschaft-im-ueberblick.html (2023, accessed 3 July 2023).

[50] Bosch-Sijtsema PM Bosch J . Plays nice with others? Multiple ecosystems, various roles and divergent engagement models. Technol Anal Strateg Manag 2015; 27: 960–974.

[51] Nickerson RC Varshney U Muntermann J . A method for taxonomy development and its application in information systems. Eur J Inf Syst 2013; 22: 336–359.

[52] Spranger J Homberg A Sonnberger M , et al. Reporting guidelines for Delphi techniques in health sciences: a methodological review. Z Evid Fortbild Qual Gesundhwes 2022; 172: 1–11. 35718726 10.1016/j.zefq.2022.04.025

[53] Braun V Clarke V . Using thematic analysis in psychology. Qual Res Psychol 2006; 3: 77–101.

[54] von Elm E Schreiber G Haupt CC . Methodische anleitung für scoping reviews (JBI-methodologie). Z Evid Fortbild Qual Gesundhwes 2019; 143: 1–7. 31296451 10.1016/j.zefq.2019.05.004

[55] Tricco AC Lillie E Zarin W , et al. PRISMA extension for scoping reviews (PRISMA-ScR): checklist and explanation. Ann Intern Med 2018; 169: 467–473. 30178033 10.7326/M18-0850

[56] Stark AL Geukes C Dockweiler C . Digital health promotion and prevention in settings: scoping review. J Med Internet Res 2022; 24: e21063. 10.2196/21063 PMC8838600 35089140

[57] Shaffer KM Turner KL Siwik C , et al. Digital health and telehealth in cancer care: a scoping review of reviews. Lancet Digit Health 2023; 5: e316–e327. 10.1016/S2589-7500(23)00049-3 PMC10124999 37100545

[58] Page MJ McKenzie JE Bossuyt PM , et al. The PRISMA 2020 statement: an updated guideline for reporting systematic reviews. Br Med J 2021; 372: n71. 10.1136/bmj.n71 PMC8005924 33782057

[59] Meyermann A Porzelt M . Hinweise zur Anonymisierung qualitativer Daten. Version 1.1. 1st ed. Frankfurt am Main: DIPF – Leibniz-Institut für Bildungsforschung und Bildungsinformation, 2014.

[60] Chapman AL Hadfield M Chapman CJ . Qualitative research in healthcare: an introduction to grounded theory using thematic analysis. J R Coll Physicians Edinb 2015; 45: 201–205. 26517098 10.4997/JRCPE.2015.305

[61] Tong A Sainsbury P Craig J . Consolidated criteria for reporting qualitative research (COREQ): a 32-item checklist for interviews and focus groups. Int J Qual Health Care 2007; 19: 349–357. 17872937 10.1093/intqhc/mzm042

[62] Davis BD Swenson A . How carequality, the Sequoia project, and eHealth exchange support the interoperable exchange of health data in the USA. J Digit Imaging 2022; 35: 812–816. 36070015 10.1007/s10278-021-00538-y PMC9450827

[63] Tikkanen R Osborn R Mossialos E , et al. International profiles of health care systems, https://www.commonwealthfund.org/sites/default/files/2020-12/International_Profiles_of_Health_Care_Systems_Dec2020.pdf (2020, accessed 15 February 2024).

[64] OECD. Health at a glance 2023: OECD indicators. Paris: OECD Publishing, 2023.

[65] Bundesministerium für Wirtschaft und Klimaschutz der Bundesrepublik Deutschland. Konsumausgaben ausgewählter Bereiche der Gesundheitswirtschaft auf dem ersten und zweiten Gesundheitsmarkt im Jahr 2022, https://de.statista.com/statistik/daten/studie/553326/umfrage/konsumausgaben-der-gesundheitswirtschaft-auf-dem-ersten-und-zweiten-gesundheitsmarkt/ (2023, accessed 2 April 2024).

[66] Zhang L Wang X Xiao H , et al. Governance mechanisms for chronic disease diagnosis and treatment systems in the post-pandemic era. Front Public Health 2022; 10: 1023022. 36582374 10.3389/fpubh.2022.1023022 PMC9792788

[67] Monti A . Monetisierungs- und preispolitik in der plattformökonomie – implikationen und handlungsempfehlungen. In: Stummeyer C Raab A Behm ME (eds) Plattformökonomie im gesundheitswesen. 1st ed. Wiesbaden: Springer Fachmedien Wiesbaden, 2023, pp.201–220.

[68] Inoue Y . Winner-takes-all or co-evolution among platform ecosystems: a look at the competitive and symbiotic actions of complementors. Sustainability 2019; 11: 726.

[69] Tudor Hart J . The inverse care law. Lancet 1971; 297: 405–412. 10.1016/s0140-6736(71)92410-x 4100731

[70] Li J-PO Liu H Ting DSJ , et al. Digital technology, tele-medicine and artificial intelligence in ophthalmology: a global perspective. Prog Retin Eye Res 2021; 82: 100900. 32898686 10.1016/j.preteyeres.2020.100900 PMC7474840

[71] Visconti R M Morea D . Healthcare digitalization and pay-for-performance incentives in smart hospital project financing. Int J Environ Res Public Health 2020; 17: 2318. 32235517 10.3390/ijerph17072318 PMC7177756

[72] Andrusko M Haber D Wolf D , et al. Payvidors, unbundled: opportunities in healthcare Fintech, https://a16z.com/2022/06/01/payvidors-unbundled-opportunities-in-healthcare-fintech/ (2022, accessed 26 June 2023).

[73] Chipman A . Government will ringfence money for health tech – Health Secretary, https://www.digitalhealth.net/2023/06/government-will-ringfence-money-for-health-tech-health-secretary/ (2023, accessed 9 September 2023).

[74] Bader N Sönnichsen B . Elektronische Patientenakte: Lauterbachs digitale Aufholjagd. Der Bundestag hat eines der wichtigsten Vorhaben von Gesundheitsminister Lauterbach beschlossen: Ab 2025 sollen alle eine elektronische Patientenakte bekommen. Kritikern gehen die Pläne viel zu weit, https://www.tagesschau.de/inland/innenpolitik/elektronische-patientenakte-112.html (2023, accessed 22 December 2023).

[75] Seifert A . Nur wenige Deutsche nutzen bislang die elektronische Patientenakte, https://www.mdr.de/nachrichten/deutschland/gesellschaft/wenige-elektronische-patientenakten-bisher-100.html (2024, accessed 5 April 2024).

[76] Curioso WH . Building capacity and training for digital health: challenges and opportunities in Latin America. J Med Internet Res 2019; 21: e16513. 10.2196/16513 PMC6939247 31850849

[77] Siribaddana P Hewapathirana R Sahay S , et al. ‘Hybrid doctors’ can fast track the evolution of a sustainable e-health ecosystem in low resource contexts: the Sri Lankan experience. Stud Health Technol Inform 2019; 264: 1356–1360. 31438147 10.3233/SHTI190448

[78] Sibuyi I-N de La Harpe R Nyasulu P . A stakeholder-centered mHealth implementation inquiry within the digital health innovation ecosystem in South Africa: momConnect as a demonstration case. JMIR mHealth uHealth 2022; 10: e18188. 10.2196/18188 PMC9247812 35708756

