# Supplementary material for: How to establish digital health ecosystems from the perspective of health service-organizations: A taxonomy developed based on expert interviews conducted as modified Delphi approach
Source: Digit Health. 2024 Aug 8;10:20552076241271890. doi: 10.1177/20552076241271890 (PMC11311194; doi:10.1177/20552076241271890)
Supplement: sj-docx-1-dhj-10.1177_20552076241271890 - Supplemental material for How to establish digital health ecosystems from the perspective of health service-organizations: A taxonomy developed based on expert interviews conducted as modified Delphi approach [file sj-docx-1-dhj-10.1177_20552076241271890.docx]

**Original Research – Supplementary Methods 1 – Literature review reporting checklist**

# How to establish digital health ecosystems from the perspective of health service-organizations: a taxonomy developed based on expert interviews conducted as modified Delphi approach

Robin Huettemann^1,5^, Benedict Sevov^1,6^, Sven Meister^2,3,7^, Leonard Fehring^1,4,8,*^

Affiliations:

1: Faculty of Health, School of Medicine, Witten/Herdecke University, Witten, Germany. *[Primary affiliation]*

2: Healthcare Informatics, Faculty of Health, School of Medicine, Witten/Herdecke University, Witten, Germany. *[Primary affiliation]*

3: Department Healthcare, Fraunhofer Institute for Software and Systems Engineering ISST, Dortmund, Germany.

4: Gastroenterology, HELIOS University Hospital Wuppertal, University Witten/Herdecke, Wuppertal, Germany.

5: ORCID: 0000-0003-3908-3029

6: ORCID: 0009-0000-2959-2394

7: ORCID: 0000-0003-0522-986X

8: ORCID: 0000-0002-3322-3724

[**www.twitter.com/DrSvenMeister**](https://urldefense.com/v3/__http:/www.twitter.com/DrSvenMeister__;!!EIXh2HjOrYMV!fk9QKSiXlI79A1YAxO_RN7XaedQ7N0xztTjsz2ZuMW3gNNoPy4ePqHxUFJFObUQgXT6j9Kltsos1daVtvdFKX-OSZK4MKzra$)

* Corresponding author:

**Leonard Fehring**

**Address**

Witten/Herdecke University

School of Medicine

Faculty of Health

Alfred-Herrhausen-Strasse 50

58448 Witten

Germany

Email leonard.fehring@uni-wh.de

Phone +49 157 85520426

## Supplementary Methods 1. Reporting of the literature review along the 27-item checklist: ‘PRISMA 2020 Checklist’ ^1^.

| **Section and topic** | **Items** | **Checklist item description** | **Item reporting** | **Location of item reporting** |
| --- | --- | --- | --- | --- |
| **Title** | | | | |
| **Title** | **1** | Identify the report as a systematic review. | PRISMA approach is not mentioned in the title but abstract. |  |
| **Abstract** | | | | |
| **Abstract** | **2** | See the PRISMA 2020 for Abstracts checklist. | PRISMA approach is not mentioned in the abstract, as a literature review guided by the steps for PRISMA ScRs was conducted. |  |
| **Introduction** | | | | |
| **Rationale** | **3** | Describe the rationale for the review in the context of existing knowledge. | The rationale was to leverage findings from previous research related to the context of the research question in this study (e.g., from other countries or previous stages in the evolution of digital health ecosystems) to reflect upon the latest scientific knowledge. |  |
| **Objectives** | **4** | Provide an explicit statement of the objective(s) or question(s) the review addresses. |  | Introduction chapter. |
| **Methods** | | | | |
| **Eligibility criteria** | **5** | Specify the inclusion and exclusion criteria for the review and how studies were grouped for the syntheses. |  | Methods chapter and Figure 1. |
| **Information sources** | **6** | Specify all databases, registers, websites, organizations, reference lists and other sources searched or consulted to identify studies. Specify the date when each source was last searched or consulted. |  |  |
| **Search strategy** | **7** | Present the full search strategies for all databases, registers and websites, including any filters and limits used. |  |  |
| **Selection process** | **8** | Specify the methods used to decide whether a study met the inclusion criteria of the review, including how many reviewers screened each record and each report retrieved, whether they worked independently, and if applicable, details of automation tools used in the process. |  |  |
| **Data collection process** | **9** | Specify the methods used to collect data from reports, including how many reviewers collected data from each report, whether they worked independently, any processes for obtaining or confirming data from study investigators, and if applicable, details of automation tools used in the process. |  |  |
| **Data items** | **10a** | List and define all outcomes for which data were sought. Specify whether all results that were compatible with each outcome domain in each study were sought (e.g., for all measures, time points, analyses), and if not, the methods used to decide which results to collect. | Non-applicable for this study, given that the outcomes of the included studies were not directly compared to each other. The comparability of outcomes across included studies is limited, given the different stakeholders included, geographic focuses, methodologies used, and timing (pre- versus post-COVID-19 pandemic). The relevant outcomes of each study were synthesized and aggregated as a priori codes to reflect the existing literature linked to the research questions of this study. The derived a priori codes were, as part of this study, confirmed, iteratively revised, and supplemented through primary data from expert interviews collected in this study. Thus, the outcomes of the included studies were not presented in a consolidated form as the final outcomes of this study. |  |
|  | **10b** | List and define all other variables for which data were sought (e.g., participant and intervention characteristics, funding sources). Describe any assumptions made about any missing or unclear information. |  | Methods chapter and Supplementary Results 1. |
| **Study risk of bias assessment** | **11** | Specify the methods used to assess risk of bias in the included studies, including details of the tool(s) used, how many reviewers assessed each study and whether they worked independently, and if applicable, details of automation tools used in the process. |  | Methods chapter. |
| **Effect measures** | **12** | Specify for each outcome the effect measure(s) (e.g., risk ratio, mean difference) used in the synthesis or presentation of results. | Non-applicable for this study, as effect strengths have not been evaluated given the qualitative research methodology employed in this study and in most of the included studies. |  |
| **Synthesis methods** | **13a** | Describe the processes used to decide which studies were eligible for each synthesis (e.g., tabulating the study intervention characteristics and comparing against the planned groups for each synthesis (item 5)). |  | Methods chapter, Figure 1, and Supplementary Results 1. |
|  | **13b** | Describe any methods required to prepare the data for presentation or synthesis, such as handling of missing summary statistics, or data conversions. | Non-applicable for this study, as all included studies were complete along the defined comprehensive overview dimensions. |  |
|  | **13c** | Describe any methods used to tabulate or visually display results of individual studies and syntheses. |  | Methods chapter, Figure 1, and Supplementary Results 1. |
|  | **13d** | Describe any methods used to synthesize results and provide a rationale for the choice(s). If meta-analysis was performed, describe the model(s), method(s) to identify the presence and extent of statistical heterogeneity, and software package(s) used. |  | Methods chapter. |
|  | **13e** | Describe any methods used to explore possible causes of heterogeneity among study results (e.g., subgroup analysis, meta regression). | Non-applicable for this study, due to the qualitative research methodology used in this study and in (most) of the included studies. |  |
|  | **13f** | Describe any sensitivity analyses conducted to assess robustness of the synthesized results |  |  |
| **Reporting bias assessment** | **14** | Describe any methods used to assess risk of bias due to missing results in a synthesis (arising from reporting biases). | Some potential limitations occur from the used search strategy, potentially limiting the scope of included studies to the used search terms, search engine, and language. To mitigate the risk of this bias, recommended studies from related fields (e.g., (business) platform ecosystem) were included, if eligible following the defined screening criteria. |  |
| **Certainty assessment** | **15** | Describe any methods used to assess certainty (or confidence) in the body of evidence for an outcome. | Non-applicable for this study, given the qualitative research methodology of this study and (most) of the included studies. |  |
| **Results** | | | | |
| **Study selection** | **16a** | Describe the results of the search and selection process, from the number of records identified in the search to the number of studies included in the review, ideally using a flow diagram. |  | Methods chapter and Figure 1. |
|  | **16b** | Cite studies that might appear to meet the inclusion criteria, but which were excluded, and explain why they were excluded. | These studies are available from the corresponding author (LF) on reasonable request. |  |
| **Study characteristics** | **17** | Cite each included study and present its characteristics. |  | Supplementary Results 1. |
| **Risk of bias in studies** | **18** | Present assessments of risk of bias for each included study. | The risk of bias due to the PRIMSA-approach for scoping reviews in this study emerges from the search strategy to identify included studies (item 14), rather than from the included studies’ findings itself. In this study, the literature scoping review is embedded in a taxonomy development methodology as overarching research approach. In this context, the derived a priori codes representing the initial taxonomy, were as part of this study confirmed, or iteratively revised and supplement through primary data from expert interviews collected, deriving the final taxonomy as findings of this study. Thus, the risk of bias in the included studies’ findings was mitigated by not presenting the literature review findings in a consolidated form as final findings of this study. |  |
| **Results of individual studies** | **19** | For all outcomes, present, for each study: (a) summary statistics for each group (where appropriate) and (b) an effect estimates and its precision (e.g., confidence/credible interval), ideally using structured tables or plots. |  | Discussion chapter. |
| **Results of syntheses** | **20a** | For each synthesis, briefly summarize the characteristics and risk of bias among contributing studies. |  | Discussion chapter and Supplementary Results 1. |
|  | **20b** | Present results of all statistical syntheses conducted. If meta-analysis was done, present for each the summary estimate and its precision (e.g., confidence/credible interval) and measures of statistical heterogeneity. If comparing groups, describe the direction of the effect. | Non-applicable for this study, given the qualitative research methodology of this study and (most) of the included studies. |  |
|  | **20c** | Present results of all investigations of possible causes of heterogeneity among study results. |  |  |
|  | **20d** | Present results of all sensitivity analyses conducted to assess the robustness of the synthesized results. |  |  |
| **Reporting biases** | **21** | Present assessments of risk of bias due to missing results (arising from reporting biases) for each synthesis assessed. | Given the research scope of this study does not include any medical evidence this risk is limited. However, given that the research field around digital health ecosystems is relatively new, the body of available literature is limited. |  |
| **Certainty of evidence** | **22** | Present assessments of certainty (or confidence) in the body of evidence for each outcome assessed. | Non-applicable for this study, given the qualitative research methodology nature of this study and (most) of the included studies. |  |
| **Discussion** | | | | |
| **Discussion** | **23a** | Provide a general interpretation of the results in the context of other evidence. |  | Results and discussion chapters. |
|  | **23b** | Discuss any limitations of the evidence included in the review. |  |  |
|  | **23c** | Discuss any limitations of the review processes used. | The search strategy closely aligned with the research question, potentially ignores studies from related fields. Please refer to item 14 for applied mitigation measures. |  |
|  | **23d** | Discuss implications of the results for practice, policy, and future research. |  | Discussion chapter. |
| **Other information** | | | | |
| **Registration and protocol** | **24a** | Provide registration information for the review, including register name and registration number, or state that the review was not registered. | Review was not registered. |  |
|  | **24b** | Indicate where the review protocol can be accessed, or state that a protocol was not prepared. | The search protocol is available from the corresponding author (LF) on reasonable request. |  |
|  | **24c** | Describe and explain any amendments to information provided at registration or in the protocol. | The search protocol provides information on the date of the search, the databases used, the applied search strategy (e.g., abstract/title search and MeSH Terms), the filters applied in the database automation tool, and the number of records. |  |
| **Support** | **25** | Describe sources of financial or non-financial support for the review, and the role of the funders or sponsors in the review. |  | Declaration statements on ‘funding’ and ‘conflict of interest’. |
| **Competing interests** | **26** | Declare any competing interests of review authors. |  |  |
| **Availability of data, code, and other materials** | **27** | Report which of the following are publicly available and where they can be found: template data collection forms; data extracted from included studies; data used for all analyses; analytic code; any other materials used in the review. | All additional information is available from the corresponding author (LF) on reasonable request. | Supplementary Results 1 and 3. |

Supplementary Material References

1. Page MJ, McKenzie JE, Bossuyt PM, et al. The PRISMA 2020 statement: an updated guideline for reporting systematic reviews. *BMJ* 2021; 372: n71.
