# Supplementary material for: How to establish digital health ecosystems from the perspective of health service-organizations: A taxonomy developed based on expert interviews conducted as modified Delphi approach
Source: Digit Health. 2024 Aug 8;10:20552076241271890. doi: 10.1177/20552076241271890 (PMC11311194; doi:10.1177/20552076241271890)
Supplement: sj-docx-2-dhj-10.1177_20552076241271890 - Supplemental material for How to establish digital health ecosystems from the perspective of health service-organizations: A taxonomy developed based on expert interviews conducted as modified Delphi approach [file sj-docx-2-dhj-10.1177_20552076241271890.docx]

**Original Research – Supplementary Methods 2 – Interview guide (Questionaire)**

# How to establish digital health ecosystems from the perspective of health service-organizations: a taxonomy developed based on expert interviews conducted as modified Delphi approach

Robin Huettemann^1,5^, Benedict Sevov^1,6^, Sven Meister^2,3,7^, Leonard Fehring^1,4,8,*^

Affiliations:

1: Faculty of Health, School of Medicine, Witten/Herdecke University, Witten, Germany. *[Primary affiliation]*

2: Healthcare Informatics, Faculty of Health, School of Medicine, Witten/Herdecke University, Witten, Germany. *[Primary affiliation]*

3: Department Healthcare, Fraunhofer Institute for Software and Systems Engineering ISST, Dortmund, Germany.

4: Gastroenterology, HELIOS University Hospital Wuppertal, University Witten/Herdecke, Wuppertal, Germany.

5: ORCID: 0000-0003-3908-3029

6: ORCID: 0009-0000-2959-2394

7: ORCID: 0000-0003-0522-986X

8: ORCID: 0000-0002-3322-3724

[**www.twitter.com/DrSvenMeister**](https://urldefense.com/v3/__http:/www.twitter.com/DrSvenMeister__;!!EIXh2HjOrYMV!fk9QKSiXlI79A1YAxO_RN7XaedQ7N0xztTjsz2ZuMW3gNNoPy4ePqHxUFJFObUQgXT6j9Kltsos1daVtvdFKX-OSZK4MKzra$)

* Corresponding author:

**Leonard Fehring**

**Address**

Witten/Herdecke University

School of Medicine

Faculty of Health

Alfred-Herrhausen-Strasse 50

58448 Witten

Germany

Email leonard.fehring@uni-wh.de

Phone +49 157 85520426

## Supplementary Methods 2. Interview guide, including five open and three one-to-five-point Likert scale-based questions to collect primary data through semi-structured qualitative expert interviews (translated into English).

The questionnaire is structured along five sections

1. **Introduction and definition**
2. **Expected value-adds**
3. **Preferred participation roles**
4. **Required capabilities including potential capability gaps**

*Depending on the Delphi round the interview is conducted in, only questions associated with the corresponding Delphi round 1 or 2 are relevant.*

**Section 1: Introduction and definition**

- Please answer the following questions from the perspective of the health service-organization group you are most familiar or associated with: 'Public payer', 'private insurer', 'healthcare service provider', or 'innovator/start-up'
- ***Definition digital health ecosystems***

*‘Digital health ecosystems are citizen-facing online applications (apps) such as mobile apps and web interfaces, which integrate, complement, and facilitate access to various digital and in-person (professional-personal) health services along the health journey. Typically, these services are provided by one or multiple health service-organizations on the supply side (e.g., healthcare service-organizations’, public payers, private insurers). These health service-organizations share the common vision of improving citizens’ well-being while enabling the efficient delivery of health services and trusted interactions with citizens on the demand side. This is orchestrated by one health service-organization in the role of a leader, operating within the policies/regulations and data/technology infrastructure standards set by governmental policymakers.’*

**Section 2:** **Expected value-adds**

- ***1) Delphi round 1*** — What value-adds do you expect from participating in digital health ecosystems? Do you agree with the presented expected value-adds from participating in digital health ecosystems derived from the literature? Would you add or deprioritize any?
  - **2)** How would you rate the relevance of each of the named value-adds to your stakeholder group?

(Please rate using a one-to-five-point Likert scale, with one being least relevant and five being most relevant)

- ***1) Delphi round 2*** — Do you agree with the presented expected value-adds from participating in digital health ecosystems, derived from the literature and previous interviews? Would you add or deprioritize any?
  - **2)** How would you rate the relevance of each of the named value-adds to your stakeholder group? Do you agree with the presented ratings derived from previous interviews, and how would you adapt them?)

(Please rate using a one-to-five-point Likert scale, with one being least relevant and five being most relevant)

**Section 3: Preferred participation roles**

- ***3) Delphi round 1*** — What role(s) do you expect to emerge in digital health ecosystems? Do you agree with the presented expected emerging roles in digital health ecosystems derived from the literature? Would you add or deprioritize any?
  - **4)** How would you rate the relevance of the expected emerging roles to your stakeholder group?

(Please rate using a one-to-five-point Likert scale, with one being least relevant and five being most relevant)

- ***3) Delphi round 2*** — Do you agree with the presented expected emerging roles in digital health ecosystems derived from the literature and previous interviews? Would you add or deprioritize any?
  - **4)** How would you rate the relevance of the expected emerging roles to your stakeholder group? Do you agree with the presented ratings derived from previous interviews, how would you adapt them?

(Please rate using a one-to-five-point Likert scale, with one being least relevant and five being most relevant)

**Section 4: Required capabilities including potential capability gaps**

- ***5) Delphi round 1*** — What are the required capabilities to participate in digital health ecosystems? Do you agree with the presented required capabilities to participate in digital health ecosystems derived from the literature? Would you add, or deprioritize any?
- ***5) Delphi round 2*** — Do you agree with the presented required capabilities to participate in digital health ecosystems derived from the literature and previous interviews? Would you add or deprioritize any?
- ***6) Delphi round 1&2*** — Do you see these capabilities as relevant to every health service-organization group participating in digital ecosystems or are they especially relevant to one stakeholder complementing others?
- ***7) Delphi round 1*** — How would you rate the current position of your health service-organization group against these required capabilities?

(Please rate using a one-to-five-point Likert scale, with one being least fulfilled and five being most fulfilled)

- ***7) Delphi round 2*** — How would you rate the current position of your health service-organization group against these required capabilities? Do you agree with the presented ratings derived from previous interviews**,** how would you adapt them?

(Please rate using a one-to-five-point Likert scale, with one being least fulfilled and five being most fulfilled)

- ***8) Delphi round 1&2*** — Closing: Are there any additional aspects of relevance you would like to mention that we have not discussed yet?
