# Supplementary material for: How to establish digital health ecosystems from the perspective of health service-organizations: A taxonomy developed based on expert interviews conducted as modified Delphi approach
Source: Digit Health. 2024 Aug 8;10:20552076241271890. doi: 10.1177/20552076241271890 (PMC11311194; doi:10.1177/20552076241271890)
Supplement: sj-docx-3-dhj-10.1177_20552076241271890 - Supplemental material for How to establish digital health ecosystems from the perspective of health service-organizations: A taxonomy developed based on expert interviews conducted as modified Delphi approach [file sj-docx-3-dhj-10.1177_20552076241271890.docx]

**Original Research – Supplementary Methods 3 – Modified Delphi approach checklist**

# How to establish digital health ecosystems from the perspective of health service-organizations: a taxonomy developed based on expert interviews conducted as modified Delphi approach

Robin Huettemann^1,5^, Benedict Sevov^1,6^, Sven Meister^2,3,7^, Leonard Fehring^1,4,8,*^

Affiliations:

1: Faculty of Health, School of Medicine, Witten/Herdecke University, Witten, Germany. *[Primary affiliation]*

2: Healthcare Informatics, Faculty of Health, School of Medicine, Witten/Herdecke University, Witten, Germany. *[Primary affiliation]*

3: Department Healthcare, Fraunhofer Institute for Software and Systems Engineering ISST, Dortmund, Germany.

4: Gastroenterology, HELIOS University Hospital Wuppertal, University Witten/Herdecke, Wuppertal, Germany.

5: ORCID: 0000-0003-3908-3029

6: ORCID: 0009-0000-2959-2394

7: ORCID: 0000-0003-0522-986X

8: ORCID: 0000-0002-3322-3724

[**www.twitter.com/DrSvenMeister**](https://urldefense.com/v3/__http:/www.twitter.com/DrSvenMeister__;!!EIXh2HjOrYMV!fk9QKSiXlI79A1YAxO_RN7XaedQ7N0xztTjsz2ZuMW3gNNoPy4ePqHxUFJFObUQgXT6j9Kltsos1daVtvdFKX-OSZK4MKzra$)

* Corresponding author:

**Leonard Fehring**

**Address**

Witten/Herdecke University

School of Medicine

Faculty of Health

Alfred-Herrhausen-Strasse 50

58448 Witten

Germany

Email leonard.fehring@uni-wh.de

Phone +49 157 85520426

## Supplementary Methods 3. Reporting of the modified Delphi approach along the 15-item checklist: 'Proposed Reporting Guidelines on Delphi Techniques in the Health Sciences’ ^1^.

| **Section and topic** | **Items** | **Item reporting** |
| --- | --- | --- |
| **Title and abstract** | **1** | The use of the modified Delphi approach is mentioned in both the title and abstract. |
| **Episte-mology** | **2** | This research is more prescribed toward the ‘constructivism’ science of theory than ‘realism’ as findings are driven by experts’ experiences due to the impracticality of interviewing all available experts. Results were objectivized by using a clear agreement definition as part of the Delphi approach to trigger content saturation, leading to the discontinuation of interviewing further participants in that group. The objective was to investigate perspectives from different health service-organization groups toward the research questions, assuming that perspectives among certain groups might be more closely aligned than others, and that perspectives might vary within groups. |
| **Formal context** | **3** | The modified Delphi approach was part of a research project investigating digital health ecosystem science. At the time of conducting the research, the four authors held the following professions: Professor, University Lectures, Researchers, Physician, Psychology Student, and Research Assistants with highest credentials including ‘Professorship’, ‘Medical Doctor’, or ‘Master of Science’. The ‘Ethics Committee of the Witten/Herdecke University’ (No. S-265/2022) did not raise an objection regarding ethical and ‘General Data Protection Regulation’ concerns. For additional information, please refer to the ‘Funding’ and ‘Ethics declarations’ statements. |
| **Knowledge base** | **4** | The starting point was a literature scoping review, following the ‘PRISMA-approach for scoping reviews’ (PRISMA-ScR) ^2–4^, to identify and incorporate relevant previous research related to the research questions (Supplementary Results 1 and Supplementary Methods 1). All interviews started with a first section aimed at disclosing and aligning relevant information with the participants to establish a shared understanding and provide the necessary background knowledge. This included relevant definitions, the scope, approach, and objectives of the interviews and study (Supplementary Methods 2).  Clinical expertise was not a requirement for participation, as the study did not focus on evidence-based decision-making, such as in patient care processes. |
| **Knowledge and knowledge integration** | **5** | Potential interview participants were considered eligible experts for this study based on their years of experience in relevant professions or positions within companies or organizations affiliated with the defined groups (Supplementary Results 2). |
| **Delphi variant and role in research process** | **6** | Modified Delphi methodologies are commonly used in health-related research ^5^, while the modification used in this study is similar to prior modifications, such as the use of ‘different expert panels for each Delphi round’. The essential components of a traditional Delphi methodology remain respected, including participant anonymity, the use of a standardized interview guide, at least one repetition of interviews, and a clear agreement definition. All criteria were reported adequately ^1,6^. |
| **Sample** | **7** | All participants were recruited via LinkedIn or their professional email address, while the initial contact was established through written communication. No incentives for the participation were offered. Participants were divided into two panels per group, representing two Delphi interview rounds. In the first round, three participants of each group were interviewed. After the first Delphi round, thematic analyses were conducted on the interview data of each group to derive the interim results. Interim results per group were updated after each additional interview in the second Delphi round. Transcripts were anonymized factually to ensure that identification of individual participants would require significant effort or may be impossible ^7^. Selectively, transcripts were smoothened when appropriated (e.g., non-verbal sentences were excluded, like ‘hm’, ‘äh’, etc. — no ‘word-by-word’ approach). Participants had the opportunity to terminate the interview at any time, but none used this option once interviews had begun. |
| **Survey instrument** | **8** | The semi-structured qualitative expert interviews were conducted using an interview guide. The interview guide was structured along the research questions and combined open and one-to-five-point Likert scale-based questions to semi-quantify the results. The same interview guide was used for all interviews (Supplementary Methods 2). |
| **Delphi rounds** | **9** | A total of two Delphi rounds were conducted. In the first round, three participants from each group were interviewed. Agreement was defined as no changes in coding or rounded ratings compared to the interim results (based on first-round results and updated after each second-round interview). This definition triggered content saturation, resulting in the discontinuation of further interviews with participants in that group. Consequently, the number of participants in the second Delphi round varied across groups (Supplementary Results 2). |
| **Feedback** | **10** | Results from Delphi rounds 1 and 2 are presented separately (Supplementary Results 4). |
| **Evaluation** | **11** | Qualitative statements were coded and clustered into themes using thematic analysis, allowing for semi-quantitative evaluations. |
| **Results** | **12** | Please refer to the following flow-chart, which illustrates the steps of the modified Delphi approach: |
|  |  |  |
| **Quality of data and inter-pretation** | **13** | Firstly, the use of expert interviews comes with typical limitations, including potential bias towards the experiences of the participants. To mitigate this, experts were thoughtfully selected as participants, groups were formed, and an objective Delphi agreement definition was defined to identify content saturation, leading to the discontinuation of interviewing further participants in that group. Secondly, there is a potential for results to be skewed toward the German health system. Both might limit the generalization of the findings. |
| **Discussion and limitations of findings** | **14** | Please refer to the Discussion chapter of the manuscript for details.  In addition, researchers are relevant in Delphi approaches, as they are considered as having an active role in the analyses which is why findings might potentially be impacted by their approach. To mitigate this limitation, experienced researchers with expertise in methodology and functional knowledge were involved in the design of the modified Delphi approach used in this study (refer to item three for details on the authors’ experience). Also, only one co-author carried out all interviews to avoid any biases between the interviews due to the conduction, while two co-authors were involved in the analysis of the interview data to prevent findings being potentially influenced by individual biases. |
| **Disse-mination** | **15** | The results of the modified Delphi approach have been presented in this article entitled ‘How to establish digital health ecosystems from the perspective of health service-organizations: a taxonomy developed based on expert interviews conducted as modified Delphi approach’. The article will be presented in a peer-reviewed journal but has not been used or presented before. |

Supplementary Material References

1. Spranger J, Homberg A, Sonnberger M, et al. Reporting guidelines for Delphi techniques in health sciences: A methodological review. *Z Evid Fortbild Qual Gesundhwes* 2022; 172: 1–11.

2. Elm E von, Schreiber G and Haupt CC. Methodische Anleitung für Scoping Reviews (JBI-Methodologie). *Z Evid Fortbild Qual Gesundhwes* 2019; 143: 1–7.

3. Tricco AC, Lillie E, Zarin W, et al. PRISMA Extension for Scoping Reviews (PRISMA-ScR): Checklist and Explanation. *Ann Intern Med* 2018; 169: 467–473.

4. Mehdi M, Stach M, Riha C, et al. Smartphone and Mobile Health Apps for Tinnitus: Systematic Identification, Analysis, and Assessment. *JMIR Mhealth Uhealth* 2020; 8: e21767.

5. Boulkedid R, Abdoul H, Loustau M, et al. Using and reporting the Delphi method for selecting healthcare quality indicators: a systematic review. *PLoS One* 2011; 6: e20476.

6. Niederberger M and Spranger J. Delphi Technique in Health Sciences: A Map. *Front Public Health* 2020; 8.

7. Meyermann A and Porzelt M. *Hinweise zur Anonymisierung qualitativer Daten. Version 1.1.* 1st ed. Frankfurt am Main: DIPF | Leibniz-Institut für Bildungsforschung und Bildungsinformation, 2014.
