# Supplementary material for: How to establish digital health ecosystems from the perspective of health service-organizations: A taxonomy developed based on expert interviews conducted as modified Delphi approach
Source: Digit Health. 2024 Aug 8;10:20552076241271890. doi: 10.1177/20552076241271890 (PMC11311194; doi:10.1177/20552076241271890)
Supplement: sj-docx-4-dhj-10.1177_20552076241271890 - Supplemental material for How to establish digital health ecosystems from the perspective of health service-organizations: A taxonomy developed based on expert interviews conducted as modified Delphi approach [file sj-docx-4-dhj-10.1177_20552076241271890.docx]

**Original Research – Supplementary Methods 4 – COREQ Checklist**

# How to establish digital health ecosystems from the perspective of health service-organizations: a taxonomy developed based on expert interviews conducted as modified Delphi approach

Robin Huettemann^1,5^, Benedict Sevov^1,6^, Sven Meister^2,3,7^, Leonard Fehring^1,4,8,*^

Affiliations:

1: Faculty of Health, School of Medicine, Witten/Herdecke University, Witten, Germany. *[Primary affiliation]*

2: Healthcare Informatics, Faculty of Health, School of Medicine, Witten/Herdecke University, Witten, Germany. *[Primary affiliation]*

3: Department Healthcare, Fraunhofer Institute for Software and Systems Engineering ISST, Dortmund, Germany.

4: Gastroenterology, HELIOS University Hospital Wuppertal, University Witten/Herdecke, Wuppertal, Germany.

5: ORCID: 0000-0003-3908-3029

6: ORCID: 0009-0000-2959-2394

7: ORCID: 0000-0003-0522-986X

8: ORCID: 0000-0002-3322-3724

[**www.twitter.com/DrSvenMeister**](https://urldefense.com/v3/__http:/www.twitter.com/DrSvenMeister__;!!EIXh2HjOrYMV!fk9QKSiXlI79A1YAxO_RN7XaedQ7N0xztTjsz2ZuMW3gNNoPy4ePqHxUFJFObUQgXT6j9Kltsos1daVtvdFKX-OSZK4MKzra$)

* Corresponding author:

**Leonard Fehring**

**Address**

Witten/Herdecke University

School of Medicine

Faculty of Health

Alfred-Herrhausen-Strasse 50

58448 Witten

Germany

Email leonard.fehring@uni-wh.de

Phone +49 157 85520426

## Supplementary Methods 4. Reporting of semi-structured qualitative expert interviews along the 32-item checklist: ‘Consolidated Criteria for Reporting Qualitative Research’ (COREQ) ^1^.

| **Section and topic** | | **Items** | **Item reporting** |
| --- | --- | --- | --- |
| **Domain 1: Research team and reflexivity** | | | |
| **Personal Character-istics** | **Interviewer/ facilitator** | **1** | All interviews were conducted by the same co-author to avoid biases. |
|  | **Credentials** | **2** | The interviewer held a 'Master of Science' degree at the time of conducting the interviews. Other researchers involved as co-authors in designing and analyzing the interviews held highest credentials including 'Professorship', 'Medical Doctor', and 'Master of Science' degrees. |
|  | **Occupation** | **3** | At the time of conducting the interviews, the interviewer was a full-time PhD student acting as a research assistant. Other researchers involved as co-authors in designing and analyzing the interviews were occupied as Professor, University Lecturers, Researchers, Physician, Psychology Student, and Research Assistant. |
|  | **Gender** | **4** | The interviewer was male. |
|  | **Experience and training** | **5** | The interviewer had experience in interviewing from different perspectives: 1) The interviewer previously conducted semi-structured qualitative interviews as part of earlier research projects. 2) In a former role as a consultant, the interviewer received professional expert interview training and conducted several interviews with different senior executives and experts in the health and insurance sectors on varies topics, partially through the expert network ‘alphasights’. In addition, the interview approach was aligned with the experienced co-authors, who had conducted semi-structured qualitative interviews in several peer-reviewed publications. Their experiences and ‘lessons-learned’ have been incorporated in the design. |
| **Relation-ship with participants** | **Relationship established** | **6** | The interviewer had no prior relationship with the participants. The first contact with potential participants was via a text message established, either on Facebook, or LinkedIn. All participants have been contacted by the interviewer directly in a written way. |
|  | **Participant knowledge of the interviewer** | **7** | Some participants were aware of the interviewer was performing the interviews as part of his Ph.D. program, as they asked during the introduction section of the interviews. Others may have visited the interviewer's LinkedIn profile, from which they could have derived the interviewer was enrolled in a Ph.D. program. |
|  | **Interviewer characteristics** | **8** | The interviewer’s characteristics or intentions were not addressed during the interview process. However, the interviewer stated the scientific reason of the interviews, the overall research sequence and objective, as well as how the interviews contribute to them. Although not mentioned proactively, when questioned, the interviewer also explained the overarching goal of obtaining a Ph.D. degree. |
| **Domain 2: Study design** | | | |
| **Theoretical framework** | **Methodological orientation and theory** | **9** | The study design followed a taxonomy development methodology, combing ‘conceptual-to-empirical’ (CtE) and ‘empirical-to-conceptual’ (EtC) approaches ^2^. For empirical evidence, primary data were collected through semi-structured qualitative expert interviews using a modified Delphi approach ^2,3^. Thematic analysis was applied to analyze the interview data ^4^*,* which is referred to as a specific type of content analysis as per Braun and Clarke: ‘[…] the unit of analysis tends to be more than a word or phrase, which it typically is in content analysis.’ ^4^ The approach allows for semi-quantitative analysis of the interviews while investigating connections, patterns, and reasonings. The approach is used in the context of grounded theory ^5^. |
| **Domain 2: Research team and reflexivity** | | | |
| **Participant selection** | **Sampling** | **10** | Participant sampling was purposive, given that specific expertise was essential for this research. It was also employed to ensure an equal number of participants across health service-organization groups in Delphi round 1 and to add participants until the Delphi agreement definition was satisfied in Delphi round 2 for each health service-organization group. Potential interview participants were considered eligible experts for this study based on their years of experience in relevant professions, professional boards, or positions within companies or organizations affiliated with the defined groups (Supplementary Results 2). |
|  | **Method of approach** | **11** | The initial contact with potential participants was made by the interviewer in written communication via a text message on LinkedIn or their professional email addresses. Participation was voluntarily as well as experts had the opportunity to terminate the interview at any time and were not offered any incentives for participation. Information on the detailed text and information shared are available from the corresponding author (LF) on reasonable request. |
|  | **Sample size** | **12** | 21 experts participated in the interviews (Supplementary Results 2), excluding three test interviews that were not considered in the findings. |
|  | **Non-participation** | **13** | We approached n=39 potential experts. N=5 did not react to the initial outreach. N=9 were interested but refused to participate either given time constraints, or compliance considerations. N=4 stated their interest but were excluded given their non-eligibility as experts after clarifying their experience. Once the interviews began, no participant chose to terminate the interview. |
| **Setting** | **Setting of data collection** | **14** | Interview data was collected through online video calls. Participants were asked to choose a comfortable environment where they could be alone and free from distractions during the interview, which in all cases was either at home or work. |
|  | **Presence of non-participants** | **15** | Only the participant and the interviewer were present during the online video calls. |
|  | **Description of sample** | **16** | Detailed characteristics of each participant are disclosed (Supplementary Results 2). |
| **Data collection** | **Interview guide** | **17** | All authors participated in designing the interview guide, which was then pre-tested with three participants, both to ensure the questions were understandable and effectively addressed the research study’s objectives. The answers from these pre-tests were not recorded, respectively were not considered in the findings of this research and these participants were not re-interviewed for the final study sample. To ensure all participants had the same understanding of digital health ecosystems, the definition was clarified at the beginning of each interview. Each semi-structured qualitative interview followed the same interview guide with open-ended questions, which remained unchanged throughout all interviews. (Supplementary Methods 2)  The interviewer might ask follow-up questions within the interview. The interviewer selectively summarized statements to ensure a correct and aligned understanding. Participants spoke freely. |
|  | **Repeat interviews** | **18** | Each participant was interviewed once, and all interviews followed the same structure and interview guide. |
|  | **Audio/ visual recording** | **19** | The semi-structured qualitative interviews were voice only recorded, as gestures, volume, facial expressions, and time stamps were not relevant to answering the research questions, protecting participants’ privacy, and creating a more comfortable environment to answer the questions. Prior to data collection, written informed consent regarding participation, interview audio recording, and publication in a peer-reviewed journal was obtained from all individual experts participating in the study. |
|  | **Field notes** | **20** | No field notes were taken, as the interviews were recorded for transcription. |
|  | **Duration** | **21** | The first Delphi round took place between 24 April and 12 May 2023, followed by a phase for deriving interim results, and the second Delphi round between 12 June and 7 July 2023. They lasted between 31 and 54 minutes each (Supplementary Results 2). |
|  | **Data saturation** | **22** | As part of the coding process, two co-authors defined the codes and discussed their repetition. Content saturation was determined through the Delphi agreement definition: Once the codes and rounded ratings per stakeholder group remained unchanged after an interview in the second Delphi round, the Delphi agreement definition was satisfied, meaning data saturation was considered achieved. This led to the discontinuation of further interviews with participants in that group. Data saturation was not discussed with the participants in or after the interviews. |
|  | **Transcripts returned** | **23** | Transcripts were not systematically returned to participants, as the voice-only recordings allowed to generate accurate and detailed transcripts. However, select participants directly asked to review the transcripts, in which case they were shared for commenting or correcting.  Transcripts were anonymized factually to ensure that identification of individual participants would require significant effort or may be impossible ^6^. Selectively, transcripts were smoothened when appropriated (e.g., non-verbal sentences were excluded, like ‘hm’, ‘äh’, etc. — no ‘word-by-word’ approach). |
| **Domain 3: Analysis and findings** | | | |
| **Data analysis** | **Number of data coders** | **24** | Interview transcripts were coded by two co-authors who first familiarized themselves with the transcripts, followed by three rounds of coding. First, statements mentioned as relevant by the participants were either assigned to one of the a priori codes or supplemented as distinct new codes. Second, codes were revised, which included adding details, renaming, or merging them. Third, a second co-author reviewed the coding. Codes were either confirmed or discussed until both were confident that the coding accurately represented the interview data. |
|  | **Description of the coding tree** | **25** | The coding tree along all levels and a detailed code description are presented (Supplementary Results 3). The first level of the coding tree links to the research questions: 1) Expected value-adds, 2) preferred participation roles, and 3) required capabilities. |
|  | **Derivation of themes** | **26** | Codes were either identified as part of the literature scoping review (referred to as a priori codes of the initial taxonomy) or derived from the thematic analysis based on the interview transcripts of the semi-structured qualitative interviews. Codes were assigned to statements mentioned as relevant by the experts and either assigned to one of the a priori codes of the initial taxonomy or supplemented as distinct new codes. Thus, a priori codes were either confirmed, detailed, renamed, merged, or supplemented by distinct new ones to derive the final coding representing the taxonomy. |
|  | **Software** | **27** | MAXQDA (Version 2022.4) was used for the data analysis. |
|  | **Participant checking** | **28** | Feedback from the participants was not collected. |
| **Reporting** | **Quotations presented** | **29** | Participant quotations were utilized to provide examples for interesting results and to facilitate the discussion. Each quotation is attributed to a specific stakeholder group and labelled accordingly. |
|  | **Data and findings consistent** | **30** | Yes, the findings partially align with previous research and is mentioned in the discussion chapter of the manuscript accordingly. |
|  | **Clarity of major themes** | **31** | The Results chapter presents the key results from the qualitative expert interviews, including the major themes and exemplary codes. Supplementary Results 3 shows the coding tree, including a granular description of all themes. Semi-quantitative results are presented in Figures 2, 4, and 5, and Supplementary Results 4. Moreover, exemplary quotes are utilized in the results and discussion chapters. |
|  | **Clarity of minor themes** | **32** | Supplementary Results 3 shows the coding tree, including a granular description of all major and minor themes identified. |

Supplementary Material References

1. Tong A, Sainsbury P and Craig J. Consolidated criteria for reporting qualitative research (COREQ): a 32-item checklist for interviews and focus groups. *Int J Qual Health Care* 2007; 19: 349–357.

2. Nickerson RC, Varshney U and Muntermann J. A method for taxonomy development and its application in information systems. *European Journal of Information Systems* 2013; 22: 336–359.

3. Spranger J, Homberg A, Sonnberger M, et al. Reporting guidelines for Delphi techniques in health sciences: A methodological review. *Z Evid Fortbild Qual Gesundhwes* 2022; 172: 1–11.

4. Braun V and Clarke V. Using thematic analysis in psychology. *Qualitative Research in Psychology* 2006; 3: 77–101.

5. Chapman AL, Hadfield M and Chapman CJ. Qualitative research in healthcare: an introduction to grounded theory using thematic analysis. *J R Coll Physicians Edinb* 2015; 45: 201–205.

6. Meyermann A and Porzelt M. *Hinweise zur Anonymisierung qualitativer Daten. Version 1.1.* 1st ed. Frankfurt am Main: DIPF | Leibniz-Institut für Bildungsforschung und Bildungsinformation, 2014.
