# Supplementary material for: How to establish digital health ecosystems from the perspective of health service-organizations: A taxonomy developed based on expert interviews conducted as modified Delphi approach
Source: Digit Health. 2024 Aug 8;10:20552076241271890. doi: 10.1177/20552076241271890 (PMC11311194; doi:10.1177/20552076241271890)
Supplement: sj-docx-5-dhj-10.1177_20552076241271890 - Supplemental material for How to establish digital health ecosystems from the perspective of health service-organizations: A taxonomy developed based on expert interviews conducted as modified Delphi approach [file sj-docx-5-dhj-10.1177_20552076241271890.docx]

**Original Research – Supplementary Results 1 – Included studies from the literature review**

# How to establish digital health ecosystems from the perspective of health service-organizations: a taxonomy developed based on expert interviews conducted as modified Delphi approach

Robin Huettemann^1,5^, Benedict Sevov^1,6^, Sven Meister^2,3,7^, Leonard Fehring^1,4,8,*^

Affiliations:

1: Faculty of Health, School of Medicine, Witten/Herdecke University, Witten, Germany. *[Primary affiliation]*

2: Healthcare Informatics, Faculty of Health, School of Medicine, Witten/Herdecke University, Witten, Germany. *[Primary affiliation]*

3: Department Healthcare, Fraunhofer Institute for Software and Systems Engineering ISST, Dortmund, Germany.

4: Gastroenterology, HELIOS University Hospital Wuppertal, University Witten/Herdecke, Wuppertal, Germany.

5: ORCID: 0000-0003-3908-3029

6: ORCID: 0009-0000-2959-2394

7: ORCID: 0000-0003-0522-986X

8: ORCID: 0000-0002-3322-3724

[**www.twitter.com/DrSvenMeister**](https://urldefense.com/v3/__http:/www.twitter.com/DrSvenMeister__;!!EIXh2HjOrYMV!fk9QKSiXlI79A1YAxO_RN7XaedQ7N0xztTjsz2ZuMW3gNNoPy4ePqHxUFJFObUQgXT6j9Kltsos1daVtvdFKX-OSZK4MKzra$)

* Corresponding author:

**Leonard Fehring**

**Address**

Witten/Herdecke University

School of Medicine

Faculty of Health

Alfred-Herrhausen-Strasse 50

58448 Witten

Germany

Email leonard.fehring@uni-wh.de

Phone +49 157 85520426

## Supplementary Results 1. Comprehensive tabular overview of included studies from the literature review along defined dimensions.

| **Items** | **Studies included** | **DOI/Access link** | **Authors** | **Journal** | **Year of pub-lication** | **Methodology** | **Regional focus** | **Health service-orga-nizations in scope** | **Details on health service-organizations in scope** |
| --- | --- | --- | --- | --- | --- | --- | --- | --- | --- |
| **1** | A Conceptual Model for Business Ecosystem and Implications for Future Research | 10.7763/IPEDR | Baghbadorani, M. F. & Harandi, A. | International proceedings of economics development and research | 2012 | Review/meta-analysis | Global | Yes, next to other stakeholders | Ecosystem roles in general — no focus on the health market |
| **2** | A model for sustainable, partnership-based telehealth services in rural India: An early process evaluation from Tuver village, Gujarat | 10.1371/journal.pone.0261907 | Ramanadhan, S. et al. | PLOS ONE | 2022 | Case study | India | Yes, next to other stakeholders | Local development foundations, telehealth providers, and design-build contractors |
| **3** | A Stakeholder-Centered mHealth Implementation Inquiry Within the Digital Health Innovation Ecosystem in South Africa: MomConnect as a Demonstration Case | 10.2196/18188 | Sibuyi, I.N., de la Harpe, R. & Nyasulu, P. | JMIR Mhealth Uhealth | 2022 | Qualitative primary data | South Africa | Yes, next to other stakeholders | Funders, professional nurses, health promoters, lay counselors, community health workers, and data capturers within the health care facility |
| **4** | Active and Assisted Living Ecosystem for the Elderly | 10.3390/s18041246 | Marcelino, I. et al. | Sensors | 2018 | Experimental | Portugal | Yes, next to other stakeholders | Services providers |
| **5** | An Improved Lightweight User Authentication Scheme for the Internet of Medical Things | 10.3390/s23031122 | Kim, K., Ryu, J., Lee, Y. & Won, D. | Sensors | 2018 | Quantitative primary data | Global | No | n/a |
| **6** | Annual review of public health | 10.1146/annurev-publhealth-052020-103738 | Grundy, Q. | Annual review of public health | 2022 | Review/meta-analysis | Global | Yes, next to other stakeholders | Health app developers |
| **7** | Assessing E-Health adoption readiness using diffusion of innovation theory and the role mediated by each adopter's category in a Mauritian context | 10.1093/inthealth/ihab035 | Putteeraj, M., Bhungee, N., Somanah, J. & Moty, N. | International health | 2022 | Quantitative primary data | Mauritius | Yes, as only stakeholder group | Health care workers |
| **8** | Building on the momentum: Sustaining telehealth beyond COVID-19 | 10.1177/1357633X20960638 | Thomas, E. E. et al. | Journal of telemedicine and telecare | 2022 | Review/meta-analysis | Global (UK, Australia, and US focus) | Yes, next to other stakeholders | Health care providers |
| **9** | Building the Digital Mental Health Ecosystem: Opportunities and Challenges for Mobile Health Innovators | 10.2196/27507 | Spadaro, B., Martin-Key, N. A. & Bahn, S. | Journal of medical internet research | 2021 | Other (e.g., viewpoint, perspective) | Global | Yes, next to other stakeholders | Innovators (not further defined) |
| **10** | Clinical Practice Experiences Using a Professional Diabetes Management Ecosystem During COVID | 10.1177/19322968211065778 | Merino Torres, J.F. et al. | Journal of diabetes science and technology | 2023 | Qualitative primary data | Belgium, Iberia, Germany, and Italy | Yes, as only stakeholder group | Health care professionals |
| **11** | Digital Health Care Industry Ecosystem: Network Analysis | 10.2196/37622 | Park Y., Park, S. & Lee, M. | Journal of medical internet research | 2022 | Quantitative primary data | South Korea | Yes, next to other stakeholders | Medical institutions and companies |
| **12** | Digital innovations for retinal care in diabetic retinopathy | 10.1007/s00592-022-01941-9 | Vujosevic, S., Limoli, C., Luzi, L. & Nucci, P. | Acta diabetologica | 2022 | Review/meta-analysis | Global | No | n/a |
| **13** | Digitally engaged physicians about the digital health transition | 10.1371/journal.pone.0238658 | Győrffy, Z., Radó, N. & Mesko, B. | PLOS ONE | 2020 | Qualitative primary data | Philippines, US, Australia, Nez Zealand, France, Israel, Hungary | Yes, as only stakeholder group | Digitally engaged physicians |
| **14** | e-Health Technological Ecosystems: Advanced Solutions to Support Informal Caregivers and Vulnerable Populations During the COVID-19 Outbreak | 10.1089/tmj.2020.0522 | Blasioli, E. & Hassini, E. | Telemedicine and e-health | 2022 | Review/meta-analysis | Global | Yes, next to other stakeholders | No clear differentiation |
| **15** | Enablers and obstacles to implementing remote monitoring technology in cardiac care: A report from an interactive workshop | 10.1177/1460458219892175 | Diaz-Skeete, Y., Giggins, O. M., McQuaid, D. & Beaney, P. | Health informatics journal | 2020 | Qualitative primary data | Ireland | Yes, next to other stakeholders | Clinicians, technologists, and representatives from health services |
| **16** | Governance mechanisms for chronic disease diagnosis and treatment systems in the post-pandemic era | 10.3389/fpubh.2022.1023022 | Zhang, L. et al. | Frontiers in Public Health | 2022 | Experimental | Global (China focus) | Yes, next to other stakeholders | Physical medical institutions, medical service platforms, and intelligent medical device providers — focused on chronic disease applications |
| **17** | Hybrid Doctors' Can Fast Track the Evolution of a Sustainable e-Health Ecosystem in Low Resource Contexts: The Sri Lankan Experience | 10.3233/SHTI190448 | Siribaddana, P., et al. | Studies in health technology and informatics | 2019 | Case study | Sri Lanka | Yes, as only stakeholder group | Hybrid doctors |
| **18** | Monitoring of motor and non-motor symptoms of Parkinson's disease through a mHealth platform | 10.1109/EMBC.2016.7590789 | Cancela, J. et al. | Annual International Conference of the IEEE Engineering in Medicine and Biology Society (EMBC) | 2016 | Experimental | Italy and Greece | No | n/a |
| **19** | Opportunities and Barriers to Rural Telerobotic Surgical Health Care in 2021: Report and Research Agenda from a Stakeholder Workshop | 10.1089/tmj.2021.0378 | Hansen, R. N. et al. | Telemedicine and e-health | 2021 | Qualitative primary data | US | Yes, as only stakeholder group | Surgeons specializing in colorectal surgery, electrophysiology, otolaryngology, and urology; technology experts representing computer science, engineering, robotics, and digital health; and health care experts trained in the fields of economics, law, public health, hospital administration, nursing, and health insurance |
| **20** | Patients' Technology Readiness and eHealth Literacy: Implications for Adoption and Deployment of eHealth in the COVID-19 Era and Beyond | 10.1097/CIN.0000000000000854 | Lee, W.L., Lim, Z.J., Tang, L.Y. et al. | Computers, informatics, nursing | 2021 | Quantitative primary data | Asia (Malaysia focus) | No | n/a |
| **21** | Personalized eHealth Program for Life-style Change: Results From the "Do Cardiac Health Advanced New Generated Ecosystem (Do CHANGE 2)" Randomized Controlled Trial | 10.1097/PSY.0000000000000802 | Broers, E.R., Widdershoven, J., Denollet, J. et al. | Psycho-somatic medicine | 2020 | Experimental | Spain and Netherlands | No | n/a |
| **22** | Plays nice with others? Multiple ecosystems, various roles and divergent engagement models | 10.1080/09537325.2015.1038231 | Bosch-Sijtsema, P. M. & Bosch, J. | Technology analysis and strategic management | 2015 | Case study | Global | Yes, next to other stakeholders | Ecosystem roles in general — no focus on the health market |
| **23** | Readiness for Delivering Digital Health at Scale: Lessons From a Longitudinal Qualitative Evaluation of a National Digital Health Innovation Program in the United Kingdom | 10.2196/jmir.6900 | Lennon, M. R. et al. | Journal of medical internet research | 2017 | Qualitative primary data | United Kingdom | Yes, next to other stakeholders | Key implementers and health professionals |
| **24** | Reinventing virtual care: Bridging the healthcare system and citizen silos to create an integrated future. | 10.1177/08404704211062575 | Borycki, E.M. & Kushniruk, A.W. | Healthcare management forum | 2022 | Review/meta-analysis | Global with focus on Canada | Yes, next to other stakeholders | Healthcare organizations and private initiatives |
| **25** | Roles during innovation ecosystem genesis: A literature review | 10.1016/j.techfore.2016.11.028 | Dedehayir, O., Mäkinen, S. J. & Roland, O. J. | Technological forecasting and social change | 2018 | Review/meta-analysis | Global | Yes, next to other stakeholders | Ecosystem roles in general — no focus on the health market |
| **26** | Smartphone and Mobile Health Apps for Tinnitus: Systematic Identification, Analysis, and Assessment | 10.2196/21767 | Mehdi, M. et al. | JMIR Mhealth Uhealth | 2020 | Review/meta-analysis | Global | No | n/a |
| **27** | Telehealth experiences of providers and patients who use augmentative and alternative communication | 10.1093/jamia/ocab273 | Beneteau, E., Paradiso, A. & Pratt, W. | Journal of the american medical informatics association | 2022 | Qualitative primary data | United Kingdom and US | Yes, next to other stakeholders | Clinicians |
| **28** | The Future of Spine Care Innovation-Software not Hardware: How the Digital Transformation Will Change Spine Care Delivery | 10.1097/BRS.0000000000004487 | Simpson, A.K. et al. | Spine journal | 2023 | Review/meta-analysis | Global | Yes, next to other stakeholders | Spine care stakeholders |
| **29** | The Limits of Empowerment: How to Reframe the Role of mHealth Tools in the Healthcare Ecosystem | 10.1007/s11948-019-00115-1 | Morley, J. & Floridi, L | Science and engineering ethics | 2019 | Review/meta-analysis | UK | No | n/a |
| **30** | Understanding digital health ecosystem from Australian citizens' perspective: A scoping review | 10.1371/journal.pone.0260058 | Alvandi, A. O., Bain, C. & Burstein, F. | PLOS ONE | 2021 | Review/meta-analysis | Australia | No | n/a |
| **31** | Using Co-design in Mobile Health System Development: A Qualitative Study With Experts in Co-design and Mobile Health System Development | 10.2196/27896 | Noorbergen, T. J., Adam, M. T. P., Teubner, T. & Collins, C. E. | JMIR Mhealth Uhealth | 2021 | Qualitative primary data | Focus on Oceania including Europe and North America | Yes, as only stakeholder group | Co-design method experts and mHealth system developers |
