# Supplementary material for: How to establish digital health ecosystems from the perspective of health service-organizations: A taxonomy developed based on expert interviews conducted as modified Delphi approach
Source: Digit Health. 2024 Aug 8;10:20552076241271890. doi: 10.1177/20552076241271890 (PMC11311194; doi:10.1177/20552076241271890)
Supplement: sj-docx-6-dhj-10.1177_20552076241271890 - Supplemental material for How to establish digital health ecosystems from the perspective of health service-organizations: A taxonomy developed based on expert interviews conducted as modified Delphi approach [file sj-docx-6-dhj-10.1177_20552076241271890.docx]

**Original Research – Supplementary Results 2 – Experts characteristics**

# How to establish digital health ecosystems from the perspective of health service-organizations: a taxonomy developed based on expert interviews conducted as modified Delphi approach

Robin Huettemann^1,5^, Benedict Sevov^1,6^, Sven Meister^2,3,7^, Leonard Fehring^1,4,8,*^

Affiliations:

1: Faculty of Health, School of Medicine, Witten/Herdecke University, Witten, Germany. *[Primary affiliation]*

2: Healthcare Informatics, Faculty of Health, School of Medicine, Witten/Herdecke University, Witten, Germany. *[Primary affiliation]*

3: Department Healthcare, Fraunhofer Institute for Software and Systems Engineering ISST, Dortmund, Germany.

4: Gastroenterology, HELIOS University Hospital Wuppertal, University Witten/Herdecke, Wuppertal, Germany.

5: ORCID: 0000-0003-3908-3029

6: ORCID: 0009-0000-2959-2394

7: ORCID: 0000-0003-0522-986X

8: ORCID: 0000-0002-3322-3724

[**www.twitter.com/DrSvenMeister**](https://urldefense.com/v3/__http:/www.twitter.com/DrSvenMeister__;!!EIXh2HjOrYMV!fk9QKSiXlI79A1YAxO_RN7XaedQ7N0xztTjsz2ZuMW3gNNoPy4ePqHxUFJFObUQgXT6j9Kltsos1daVtvdFKX-OSZK4MKzra$)

* Corresponding author:

**Leonard Fehring**

**Address**

Witten/Herdecke University

School of Medicine

Faculty of Health

Alfred-Herrhausen-Strasse 50

58448 Witten

Germany

Email leonard.fehring@uni-wh.de

Phone +49 157 85520426

## Supplementary Results 2. Detailed characteristics of the 21 experts participating in the semi-structured qualitative expert interviews.

The first Delphi round took place between 24 April and 12 May 2023, followed by a phase for deriving interim results, and the second Delphi round between 12 June and 7 July 2023.

| **Expert** | **Stakeholder group** | **Organization** | **Position** | **Delphi round** | **Interview time,** in minutes |
| --- | --- | --- | --- | --- | --- |
| **E1** | **Payer** | Public payer IT platform provider | Managing Director (CEO) | 1 | 39 |
| **E2** | **Payer** | Digital unit of leading German payer | Digital Strategy Developer | 1 | 54 |
| **E11** | **Payer** | Digital unit of leading German payer | Digital Health Expert | 1 | 51 |
| **E21** | **Payer** | Digital strategy consultancy for payer | Co-Founder and CEO | 2 | 41 |
| **E3** | **Insurer** | Digital health ecosystem consultancy | Founder & Managing Director (CEO) | 1 | 37 |
| **E8** | **Insurer** | Digital health entity of leading German health insurer | Innovation Manager | 1 | 46 |
| **E10** | **Insurer** | Digital health entity of globally leading multi-line insurer based in Germany | Head of Innovation Management | 1 | 34 |
| **E13** | **Insurer** | Leading health insurer in Germany | Head of Program Management Digital Health Services | 2 | 36 |
| **E6** | **Provider** | Digital health innovation consultancy for hospitals | Founder & CEO | 1 | 42 |
| **E9** | **Providers** | Leading German hospital chain | Senior Project Leader Digital Health | 1 | 51 |
| **E12** | **Provider** | University hospital of a large German city | Chief Digital Officer | 1 | 43 |
| **E15** | **Provider** | University hospital of a large German city | Head of Digital Clinician Science | 2 | 33 |
| **E17** | **Provider** | IT strategy advisory for hospitals | Senior Project Manager | 2 | 44 |
| **E18** | **Provider** | University hospital of a large German city | Head Physician for a certain Clinical Pattern | 2 | 33 |
| **E19** | **Provider** | Leading German hospital chain | Head of Digital Health, Data and Analytics | 2 | 31 |
| **E4** | **Innovator** | Hospital rating and comparison platform | Business Development and Partnerships | 1 | 41 |
| **E5** | **Innovator** | Patient-service-provider matching platform | Chief Business Development Officer | 1 | 46 |
| **E7** | **Innovator** | Digital health ecosystem start-up consultancy and supervisory board member of three digital health start-ups | Founder & CEO | 1 | 39 |
| **E14** | **Innovator** | Digital health ecosystem start-up consultancy | Senior Market Access Consultant | 2 | 52 |
| **E16** | **Innovator** | Digital mental health platform | Co-Founder & CEO | 2 | 49 |
| **E20** | **Innovator** | Patient-service-provider matching and prevention recommendation platform | Founder & CEO | 2 | 31 |
