# Supplementary material for: How to establish digital health ecosystems from the perspective of health service-organizations: A taxonomy developed based on expert interviews conducted as modified Delphi approach
Source: Digit Health. 2024 Aug 8;10:20552076241271890. doi: 10.1177/20552076241271890 (PMC11311194; doi:10.1177/20552076241271890)
Supplement: sj-docx-7-dhj-10.1177_20552076241271890 - Supplemental material for How to establish digital health ecosystems from the perspective of health service-organizations: A taxonomy developed based on expert interviews conducted as modified Delphi approach [file sj-docx-7-dhj-10.1177_20552076241271890.docx]

**Original Research – Supplementary Results 3 – Final taxonomy**

# How to establish digital health ecosystems from the perspective of health service-organizations: a taxonomy developed based on expert interviews conducted as modified Delphi approach

Robin Huettemann^1,5^, Benedict Sevov^1,6^, Sven Meister^2,3,7^, Leonard Fehring^1,4,8,*^

Affiliations:

1: Faculty of Health, School of Medicine, Witten/Herdecke University, Witten, Germany. *[Primary affiliation]*

2: Healthcare Informatics, Faculty of Health, School of Medicine, Witten/Herdecke University, Witten, Germany. *[Primary affiliation]*

3: Department Healthcare, Fraunhofer Institute for Software and Systems Engineering ISST, Dortmund, Germany.

4: Gastroenterology, HELIOS University Hospital Wuppertal, University Witten/Herdecke, Wuppertal, Germany.

5: ORCID: 0000-0003-3908-3029

6: ORCID: 0009-0000-2959-2394

7: ORCID: 0000-0003-0522-986X

8: ORCID: 0000-0002-3322-3724

[**www.twitter.com/DrSvenMeister**](https://urldefense.com/v3/__http:/www.twitter.com/DrSvenMeister__;!!EIXh2HjOrYMV!fk9QKSiXlI79A1YAxO_RN7XaedQ7N0xztTjsz2ZuMW3gNNoPy4ePqHxUFJFObUQgXT6j9Kltsos1daVtvdFKX-OSZK4MKzra$)

* Corresponding author

**Leonard Fehring**

**Address**

Witten/Herdecke University

School of Medicine

Faculty of Health

Alfred-Herrhausen-Strasse 50

58448 Witten

Germany

Email leonard.fehring@uni-wh.de

Phone +49 157 85520426

## Supplementary Results 3. Final developed taxonomy including the coding tree across levels, code descriptions, and code origination.

| **Coding level 1**  (Meta-characteristics) | **Coding level 2** | **Coding level 3** | **Coding level 4** | **Code description** | **Code items** | **Code origination** (’conceptual-to-empirical (CtE)/’ ’empirical-to-conceptual’ (CtE)) |
| --- | --- | --- | --- | --- | --- | --- |
| **Expected value-adds** | **Increasing citizens’ confidence** | **Empower citizens, or relatives in making health-related decisions** |  | Increasing the knowledge and health-related decision-making ability among citizens and their relatives, including the critical reflection of service-providers' recommendations. | 1 | CtE |
|  |  | **Intensify partnership with citizens beyond episodical treatments** |  | Increasing the frequency, channels, and speed of interactions with citizens, even for less urgent topics, to foster deeper relationships along health journeys. | 2 | CtE |
|  | **Improving health journeys** | **Improve or personalize health treatments (1)** |  | Improving citizens’ actual health through personalized health approaches, also enabling quicker recoveries. | 3 | CtE |
|  |  | **Expand and differentiate the own service cooperating with third parties** |  | Expanding the range of one's own service by integrating additional third-party services, either related to the current ones or extending beyond them. | 4 | EtC |
|  |  | **Improve access to health services, data management, and prevention services (2)** |  | Improving the health experience by minimizing travel requirements, reducing waiting times, optimizing data management, improving health access, and tailoring relevant prevention services to individual needs. | 5 | CtE |
|  | **Strengthen-ing the health process economics** | **Expand the pool of served citizens** |  | Serving a higher number of citizens than currently. | 6 | CtE |
|  |  | **Increase efficiencies of workflows, reimbursement payments, or relief employees** |  | Increasing the operational workflow efficiency (e.g., paperless) internally or of ecosystem partners, through digitization and automation measures aiming to save costs, free up staff capacity, or steer reimbursement payments. | 7 | CtE |
| **Preferred participation roles** | **Orchestrator** | **Orchestrator** |  | Acting as a central organizer, connecting the stakeholders on the demand and supply sides, setting the overall vision, coordinating exchanges among all stakeholders, and typically providing the necessary platform infrastructure. | 8 | CtE |
|  |  | **Light orchestrator** |  | Compiling and coordinating services exclusively for the organization's own clients within a digital health ecosystem organized by another ‘orchestrator’. | 9 | EtC |
|  | **Complementor** | **Complementor** |  | Integrating one's own service into a digital health ecosystem targeted toward citizens. | 10 | CtE |
|  |  | **Medical complementor** |  | Improving or facilitating access to preventive, diagnostic, and treatment services within a digital health ecosystem. | 11 | EtC |
|  |  | **Financial complementor** |  | Managing the payment and reimbursement of services within a digital health ecosystem. | 12 | EtC |
|  | **Enabler** | **Enabler** |  | Integrating services targeted toward health service-organizations within a digital health ecosystem. | 13 | CtE |
|  |  | **Technical enabler** |  | Facilitating platform infrastructure development, data analytics, interface design, and digital journey design requirements within a digital health ecosystem. | 14 | EtC |
|  |  | **Regulatory enabler** |  | Accessing and processing health-related citizen data in a 'pseudo-anonymized' way to make those legally available to all health service-organizations within a digital health ecosystem. | 15 | EtC |
| **Required capabilities** | **Health market readiness** | **Market understanding** | Understanding of the regulatory/ policy and funding environment (1) | Understanding legal boundaries, including requirements for personal data processing, data access, and financial support within the given environment. | 16 | CtE |
|  |  |  | Understanding of target groups’ expectations (2) | Understanding the digital journey needs in terms of services, communication channels, usability, and design of (a) dedicated citizen target group(s). | 17 | CtE |
|  |  | **Reach and relevance** | Access to key opinion leaders and persuasive power (1) | Leveraging contacts to key health decision-makers with the power to persuade or holding a relevant position within the health network with the ability to influence. | 18 | EtC |
|  |  |  | Awareness and trust in the own service among citizens or existing client base (2) | Having awareness, popularity (brand reach), and a recognized position as a trusted health service-organization among citizens, potentially supported by a large existing client base. | 19 | CtE |
|  |  | **Value-adding service** | Clinical evidence based (digital) medical service (1) | Providing a service with proven and documented medical efficacy. | 20 | CtE |
|  |  |  | Citizen centered (monetizable or frequent interaction) service (2) | Having a proven monetization concept with a clear revenue stream, potentially through the creation of recurring client interactions. | 21 | EtC |
|  | **Organizational readiness** | **Ecosystem mind-set** | Long-term commitment, with sustainable health process economics for all health service-organizations | Demonstrating a long-term commitment to integrating as a partner into digital health ecosystems based on transparent collaboration and communication, while allowing each health service-organization to (financially) benefit sustainably (e.g., to share created value). | 22 | CtE |
|  |  |  | Openness to cooperate and to share a vision, data, governance, and standards | Embracing openness to share information and data with partners, as well as the ability to adapt to defined standards and definitions (e.g., EHR) set by digital health ecosystem partners or governmental policymakers. | 23 | EtC |
|  |  |  | Clear understanding of ecosystem role and strategy | Having a senior management with a clear strategy on the organization's own role in the digital health ecosystem, including a defined target group, understanding of capabilities required, and a roadmap to build the required capabilities, to reach the envisioned position. | 24 | CtE |
|  |  | **Employer culture** | Agility to react to changes and quick decision making | Having a digital health ecosystem responsible for making decisions within defined boundaries and the flexibility to assemble relevant organizational decision-makers efficiently to enable timely decision-making. | 25 | EtC |
|  |  |  | Human, project, and change management resources | Demonstrating the ability to effectively manage organizational change, communicate, and advocate for change among employees, as well as to attract, retain, and train the necessary talent pool. | 26 | CtE |
|  |  | **Financials** | Financial resources for investments | Allocating sufficient financial resources to invest in building the required capabilities and scaling the digital health ecosystem. | 27 | CtE |
|  | **Technology and informatic readiness** | **Frontend journey** | Easy-to-use digital frontend solution | Having a digital citizen-facing frontend interface that allows easy and intuitive navigation, without the need for additional information or instructions, tailored to the defined target group. | 28 | CtE |
|  |  | **Technical backend** | Scalable and reliable platform with modern/open API structure | Implementing a consistently available middleware platform that connects demand and supply side stakeholders, with a structured and scalable backend equipped with open and modern ‘Application Programming Interfaces’ (APIs) to facilitate the integration of health service-organizations. | 29 | CtE |
|  |  |  | Reliable data protection and cybersecurity | Preventing data misuse by ensuring the collection, storage, and sharing of data remains unexceptional within legal and digital health ecosystem defined boundaries, while constantly combating cyber incidents and attacks to build citizen trust. | 30 | CtE |
|  |  |  | Interoperability of data collection and processing | Enabling automated and digital data download, upload, and processing between external and internal sources, including real-time mapping of data across sources to individual citizens to ensure data quality and to leverage data analytics insights. | 31 | CtE |

1. Especially relevant in the primary health market I (2) Especially relevant in the secondary health market
