# Supplementary material for: How to establish digital health ecosystems from the perspective of health service-organizations: A taxonomy developed based on expert interviews conducted as modified Delphi approach
Source: Digit Health. 2024 Aug 8;10:20552076241271890. doi: 10.1177/20552076241271890 (PMC11311194; doi:10.1177/20552076241271890)
Supplement: sj-docx-8-dhj-10.1177_20552076241271890 - Supplemental material for How to establish digital health ecosystems from the perspective of health service-organizations: A taxonomy developed based on expert interviews conducted as modified Delphi approach [file sj-docx-8-dhj-10.1177_20552076241271890.docx]

**Original Research – Supplementary Results 4 – Rating results by Delphi interview rounds**

# How to establish digital health ecosystems from the perspective of health service-organizations: a taxonomy developed based on expert interviews conducted as modified Delphi approach

Robin Huettemann^1,5^, Benedict Sevov^1,6^, Sven Meister^2,3,7^, Leonard Fehring^1,4,8,*^

Affiliations:

1: Faculty of Health, School of Medicine, Witten/Herdecke University, Witten, Germany. *[Primary affiliation]*

2: Healthcare Informatics, Faculty of Health, School of Medicine, Witten/Herdecke University, Witten, Germany. *[Primary affiliation]*

3: Department Healthcare, Fraunhofer Institute for Software and Systems Engineering ISST, Dortmund, Germany.

4: Gastroenterology, HELIOS University Hospital Wuppertal, University Witten/Herdecke, Wuppertal, Germany.

5: ORCID: 0000-0003-3908-3029

6: ORCID: 0009-0000-2959-2394

7: ORCID: 0000-0003-0522-986X

8: ORCID: 0000-0002-3322-3724

[**www.twitter.com/DrSvenMeister**](https://urldefense.com/v3/__http:/www.twitter.com/DrSvenMeister__;!!EIXh2HjOrYMV!fk9QKSiXlI79A1YAxO_RN7XaedQ7N0xztTjsz2ZuMW3gNNoPy4ePqHxUFJFObUQgXT6j9Kltsos1daVtvdFKX-OSZK4MKzra$)

* Corresponding author:

**Leonard Fehring**

**Address**

Witten/Herdecke University

School of Medicine

Faculty of Health

Alfred-Herrhausen-Strasse 50

58448 Witten

Germany

Email leonard.fehring@uni-wh.de

Phone +49 157 85520426

## Supplementary Results 4. Rating results differentiated by the two Delphi interview rounds and four health service-organization groups.

***
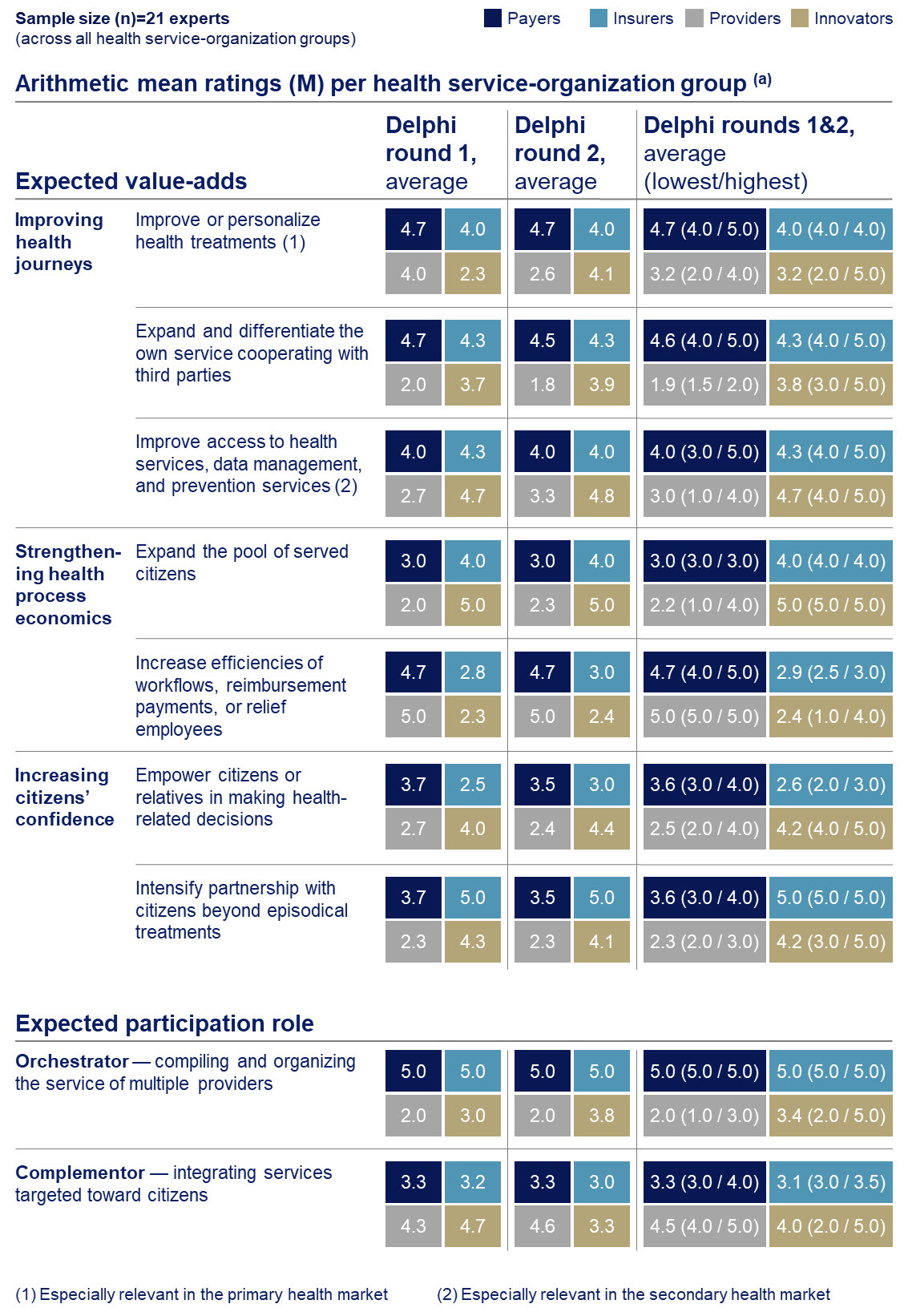
***

***
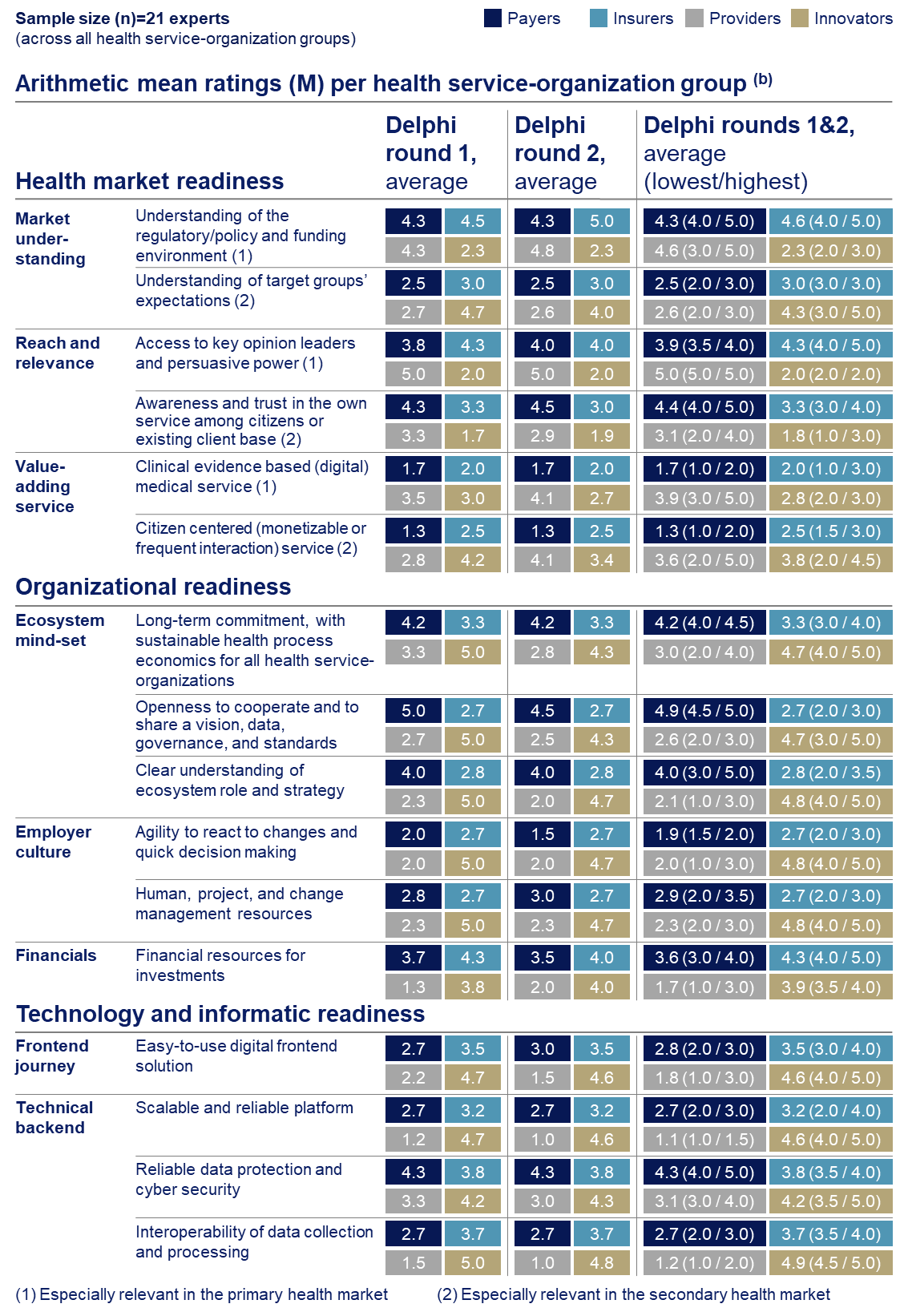
***

(a) The 21 interviewed experts were asked to rate on a one-to-five-point Likert scale. The scale ranged from 5 (most relevant) to 1 (least relevant). The arithmetic mean ratings (M) were calculated by summing the ratings of each expert within a health service-organization group and dividing by the sample size (n) of experts interviewed in that group.

(b) The 21 interviewed experts were asked to rate on a one-to-five-point Likert scale. The scale ranged from 5 (most fulfilled) to 1 (least fulfilled). The arithmetic mean ratings (M) were calculated by summing the ratings of each expert within a health service-organization group and dividing by the sample size (n) of experts interviewed in that group.
